# Erlotinib versus gemcitabine plus cisplatin as neoadjuvant treatment of stage IIIA-N2 *EGFR*-mutant non-small-cell lung cancer: final overall survival analysis of the EMERGING-CTONG 1103 randomised phase II trial

**DOI:** 10.1038/s41392-022-01286-3

**Published:** 2023-02-24

**Authors:** Wen-Zhao Zhong, Hong-Hong Yan, Ke-Neng Chen, Chun Chen, Chun-Dong Gu, Jun Wang, Xue-Ning Yang, Wei-Min Mao, Qun Wang, Gui-Bin Qiao, Ying Cheng, Lin Xu, Chang-Li Wang, Ming-Wei Chen, Xiao-Zheng Kang, Wan-Pu Yan, Ri-Qiang Liao, Jin-Ji Yang, Xu-Chao Zhang, Si-Yang Liu, Qing Zhou, Yi-Long Wu

**Affiliations:** 1 Guangdong Provincial People’s Hospital, Guangdong Academy of Medical Sciences, 510080 Guangzhou, China; 2 Peking University Cancer Hospital and Institute, 100142 Beijing, China; 3 Fujian Medical University Union Hospital, 350001 Fuzhou, China; 4 First Affiliated Hospital of Dalian Medical University, 116011 Dalian, China; 5 Peking University People’s Hospital, 100044 Beijing, China; 6 Zhejiang Cancer Hospital, 310022 Hangzhou, China; 7 Zhongshan Hospital, 200032 Shanghai, China; 8 Guangzhou Liuhuaqiao Hospital, 510000 Guangzhou, China; 9 Jilin Provincial Tumor Hospital, 130012 Changchun, China; 10 Jiangsu Cancer Institute and Hospital, 210009 Nanjing, China; 11 Tianjin Medical University Cancer Institute and Hospital, 300060 Tianjin, China; 12 First Affiliated Hospital of Xi’an Jiaotong University, 710061 Xi’an, China

**Keywords:** Cancer therapy, Lung cancer

## Abstract

EMERGING-CTONG 1103 showed improved progression-free survival (PFS) with neoadjuvant erlotinib vs. chemotherapy for patients harbouring EGFR sensibility mutations and R0 resected stage IIIA-N2 non-small cell lung cancer (NSCLC) (NCT01407822). Herein, we report the final results. Recruited patients were randomly allocated 1:1 to the erlotinib group (150 mg/day orally; neoadjuvant phase for 42 days and adjuvant phase to 12 months) or to the GC group (gemcitabine 1250 mg/m^2^ plus cisplatin 75 mg/m^2^ intravenously; 2 cycles in neoadjuvant phase and 2 cycles in adjuvant phase). Objective response rate (ORR), complete pathologic response (pCR), PFS, and overall survival (OS) were assessed along with safety. Post hoc analysis was performed for subsequent treatments after disease recurrence. Among investigated 72 patients (erlotinib, *n* = 37; GC, *n* = 35), the median follow-up was 62.5 months. The median OS was 42.2 months (erlotinib) and 36.9 months (GC) (hazard ratio [HR], 0.83; 95% confidence interval [CI], 0.47–1.47; *p* = 0.513). The 3- and 5-year OS rates were 58.6% and 40.8% with erlotinib and 55.9% and 27.6% with GC (*p*_3-y_ = 0.819, *p*_5-y_ = 0.252). Subsequent treatment was administered in 71.9% and 81.8% of patients receiving erlotinib and GC, respectively; targeted therapy contributed mostly to OS (HR, 0.35; 95% CI, 0.18–0.70). After disease progression, the ORR was 53.3%, and the median PFS was 10.9 months during the EGFR-TKI rechallenge. During postoperative therapy, grade 3 or 4 adverse events (AEs) were 13.5% in the erlotinib group and 29.4% in the GC group. No serious adverse events were observed. Erlotinib exhibited clinical feasibility for resectable IIIA-N2 NSCLC over chemotherapy in the neoadjuvant setting.

## Introduction

Despite therapeutic advances, lung cancer keeps a leading cause of cancer death worldwide^1^ and in China.^2^ Non-small-cell lung cancer (NSCLC) accounts for over 85% of lung cancer.^3^ Furthermore, most NSCLC patients are diagnosed with stage III or IV disease.^4^ Stage IIIA NSCLC is morphologically defined as a primary tumour that ipsilaterally spreads to mediastinal lymph nodes (N2).^5^ Potentially resectable IIIA-N2 NSCLC, confirmed by endobronchial ultrasound-guided biopsy, mediastinoscopy, and positron emission tomography (PET), is highly heterogeneous in terms of clinical profile, treatment modalities, and prognosis.^6^

Surgery, radiotherapy, and chemotherapy are the primary modalities of stage III NSCLC treatment. However, molecular characterisation of tumours is essential to detect specific mutations, which is even more relevant in advanced lung cancer stages. For patients with stage IV NSCLC and epidermal growth factor receptor (*EGFR*) mutations, EGFR tyrosine kinase inhibitor (EGFR-TKI) therapy is recommended as the standard first-line treatment and has shown significant survival benefit.^7–11^ Some studies have evaluated the role of neoadjuvant treatment with the EGFR-TKI erlotinib for patients with stage IIIA-N2 *EGFR*-positive NSCLC, including recent meta-analyses.^12–16^ These studies reported improvements in progression-free survival (PFS) and pathological complete response (pCR) rates in patients with mutant tumours treated with neoadjuvant EGFR-TKIs compared with chemotherapy.

The EMERGING-CTONG 1103 (ClinicalTrials.gov identifier: NCT01407822) was a multicentre (17 centres in China), open-label, phase II, randomised controlled trial of erlotinib versus gemcitabine combination with cisplatin (GC) as perioperative therapy in patients with stage IIIA-N2 NSCLC and EGFR sensibility mutations.^17^ In the first prespecified analysis (median follow-up of 25.2 months), neoadjuvant erlotinib resulted in an objective response rate (ORR) of 54.1% vs. 34.3% with GC (95% confidence interval [CI] 0.87–5.84; *p* = 0.092), but the primary endpoint of ORR was not met. The toxicity profile was also milder compared with GC. In general, neoadjuvant erlotinib was demonstrated to be clinically feasible and well-tolerated. Whether upfront targeted therapy in the perioperative setting may influence the efficacy of subsequent treatment after disease progression and further impact overall survival (OS) remains an open question. In this paper, we report the updated analysis of OS and analysed efficacy among patients receiving different treatments after disease progression and the safety profile during an extended follow-up period.

## Results

### Final and updated survival analyses

**Fig. 1:**
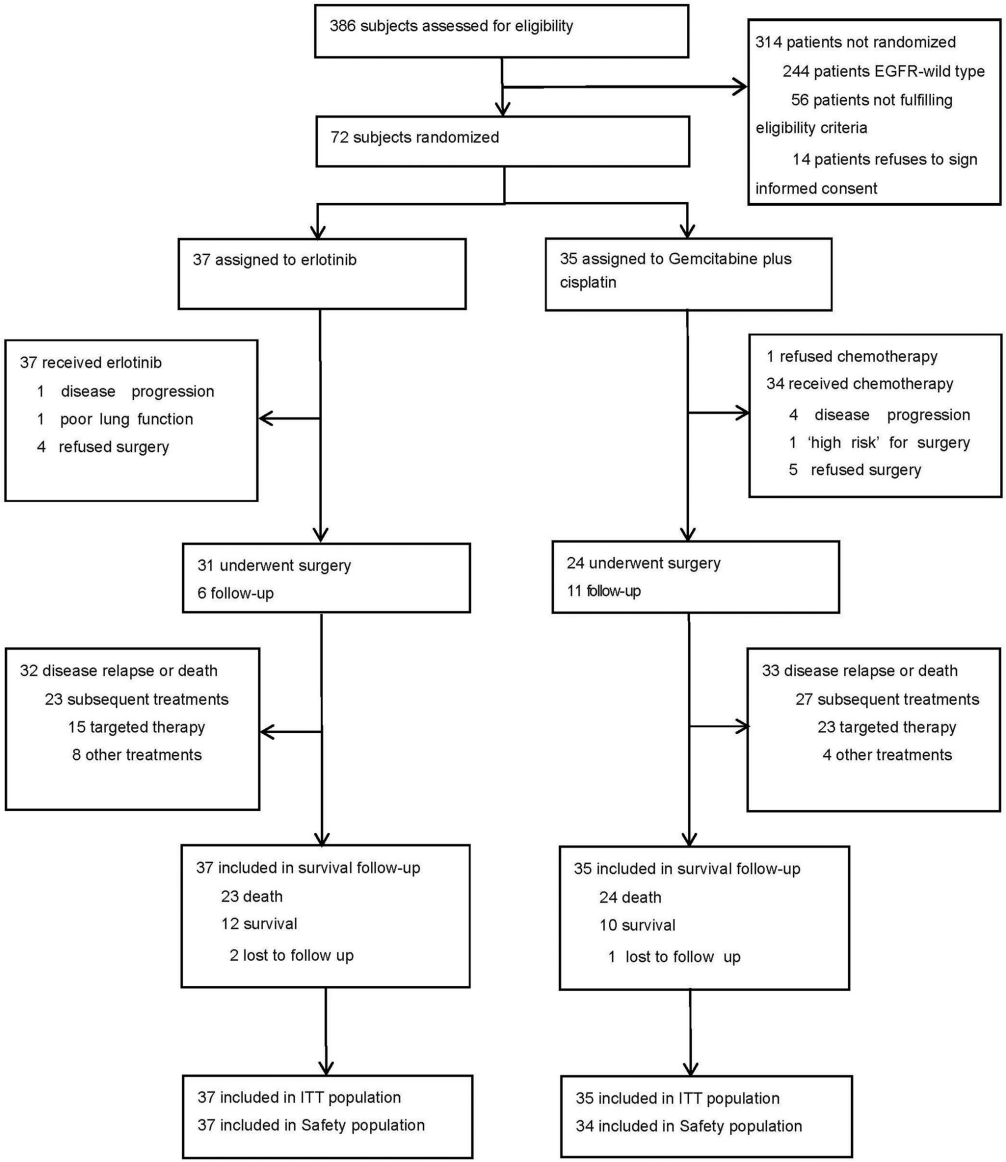
Trial profile. ITT intention-to-treat

The EMERGING-CTONG 1103 trial screened a total of 386 patients at 17 sites in China (Fig. 1). Of these, 72 patients (intention-to-treat, ITT population) were assigned to receive the erlotinib (*n* = 37) or the GC chemotherapy (*n* = 35). The study was conducted from December 5, 2011 to December 13, 2017. The demographics and baseline characteristics of patients have been reported previously^17^ and were well balanced between the two groups (Table 1). Of note, baseline clinicopathological data were well-balanced between groups.

**Table 1:** Patient demographics and clinical characteristics (intention-to-treat population)

	Erlotinib group (*n* = 37)	GC group (*n* = 35)
*Sex*		
Male	11 (29.7)	8 (22.9)
Female	26 (70.3)	27 (77.1)
*Ethnic group*		
Han	36 (97.3)	33 (94.3)
Others	1 (2.7)	2 (5.7)
Median age, years (range)	59 (32–73)	58 (33–76)
*Smoking status*		
Never smokers	29 (78.4)	31 (88.6)
Current smokers	6 (16.2)	2 (5.7)
Former smokers	2 (5.4)	2 (5.7)
*ECOG PS score*		
0	13 (35.1)	14 (40.0)
1	24 (64.9)	21 (60.0)
*Pathological type*		
Adenocarcinoma	32 (86.5)	33 (94.3)
Non-adenocarcinoma	5 (13.5)	2 (5.7)
*Preoperative staging*		
Mediastinoscopy	12 (32.4)	9 (25.7)
Bronchial ultrasound	12 (32.4)	16 (45.7)
PET/CT	13 (35.2)	10 (28.6)
*T stage*		
T1	12 (32.4)	5 (14.3)
T2	16 (43.3)	20 (57.1)
T3	7 (18.9)	10 (28.6)
T4	1 (2.7)	0 (0.0)
Tx	1 (2.7)	0 (0.0)
*N*_*2*_ *status*		
Single-station N_2_	17 (45.9)	19 (54.3)
Multi-station N_2_	20 (54.1)	16 (45.7)
*EGFR*-*activating mutations*		
Exon 19 mutation	16 (43.2)	18 (51.4)
Exon 21 mutation	21 (56.8)	17 (48.6)

Data are *n* (%) unless otherwise stated

*CT* computed tomography, *ECOG PS* Eastern Cooperative Oncology Group performance status, *EGFR* epidermal growth factor receptor, *GC* gemcitabine plus cisplatin, *PET* positron emission tomography, *x* the primary tumour could not be evaluated

**Fig. 2:**
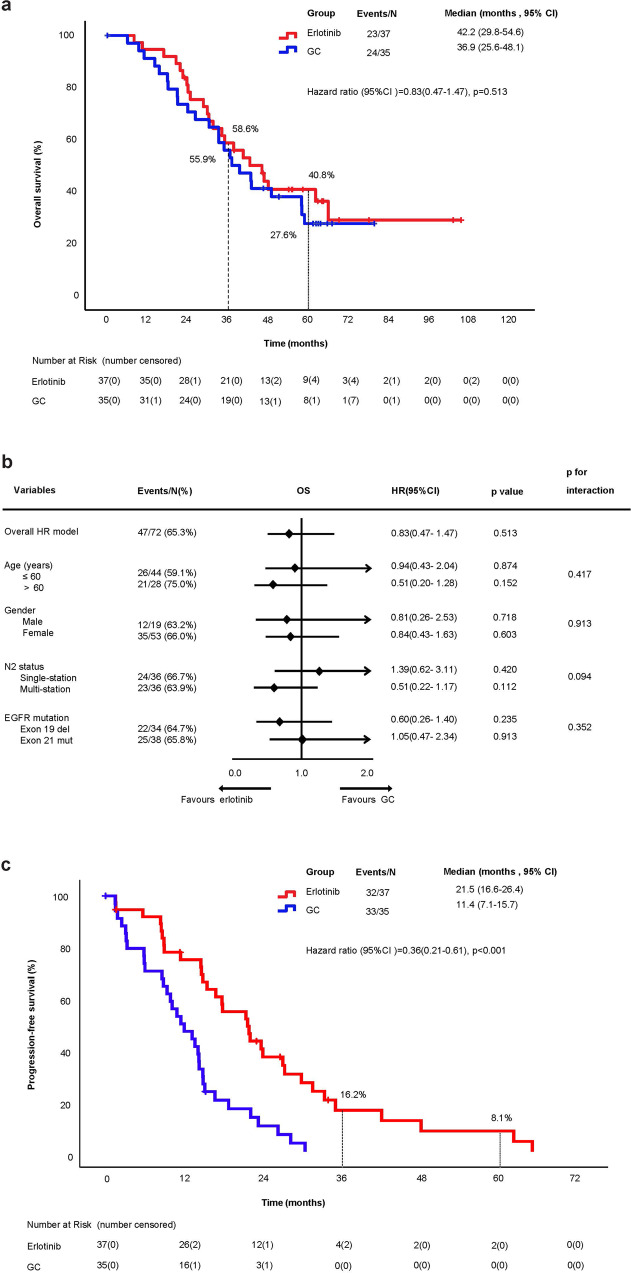
**a** Kaplan–Meier analysis of OS and **b** subgroup analysis between erlotinib and GC groups in the intention-to-treat population and **c** the update Kaplan–Meier analysis of PFS. EGFR epidermal growth factor receptor, GC gemcitabine plus cisplatin, HR hazard ratio, OS overall survival, PFS progression-free survival

During the follow-up period (median 62.5 months, interquartile range 54.8–68.7), 47 (65.3%) deaths were reported in the ITT population, including 23 patients in the erlotinib group and 24 patients in the GC group. The median OS was 42.2 months (95% CI, 29.8–54.6) in the erlotinib group and 36.9 months (95% CI, 25.6–48.1) in the GC group (hazard ratio [HR], 0.83; 95% CI, 0.47–1.47; *p* = 0.513) (Fig. 2a). At 3 years, the cumulative proportion surviving was 58.6% (95% CI, 42.5–74.7%) in the erlotinib group and 55.9% (95% CI, 39.2–72.6%) in the GC group (*p*_3-y_ = 0.819). At 5 years, the corresponding results were 40.8% (95% CI, 24.3–57.3%) and 27.6% (95% CI, 12.1–43.1%) (*p*_5-y_ = 0.252), respectively (Table 2). None of the predefined subgroup analyses for OS showed a meaningful interaction and differences according to age (≤60, >60 years), gender (male, female), N2 status (single-station, multi-station), or *EGFR* mutation type (exon 19 deletion, exon 21 L858R mutation) (Fig. 2b). Up to January 29, 2021, the median PFS was 14.7 months (95% CI, 13.4–16.0) in the overall ITT population. The median PFS was significantly prolonged with the erlotinib group (21.5 months; 95% CI, 16.6–26.4) compared to the GC group (11.4 months; 95% CI, 7.1–15.7; HR, 0.36; 95% CI, 0.21–0.61; *p* < 0.001) (Fig. 2c).

**Table 2:** OS rates at 3 and 5 years (intention-to-treat population)

	Erlotinib group (*n* = 37)	GC group (*n* = 35)	*p*
3-year OS rate	58.6 (42.5–74.7)	55.9 (39.2–72.6)	0.819
5-year OS rate	40.8 (24.3–57.3)	27.6 (12.1–43.1)	0.252

Data are % (95% CI)

*CI* confidence interval, *GC* gemcitabine plus cisplatin, *OS* overall survival

**Fig. 3:**
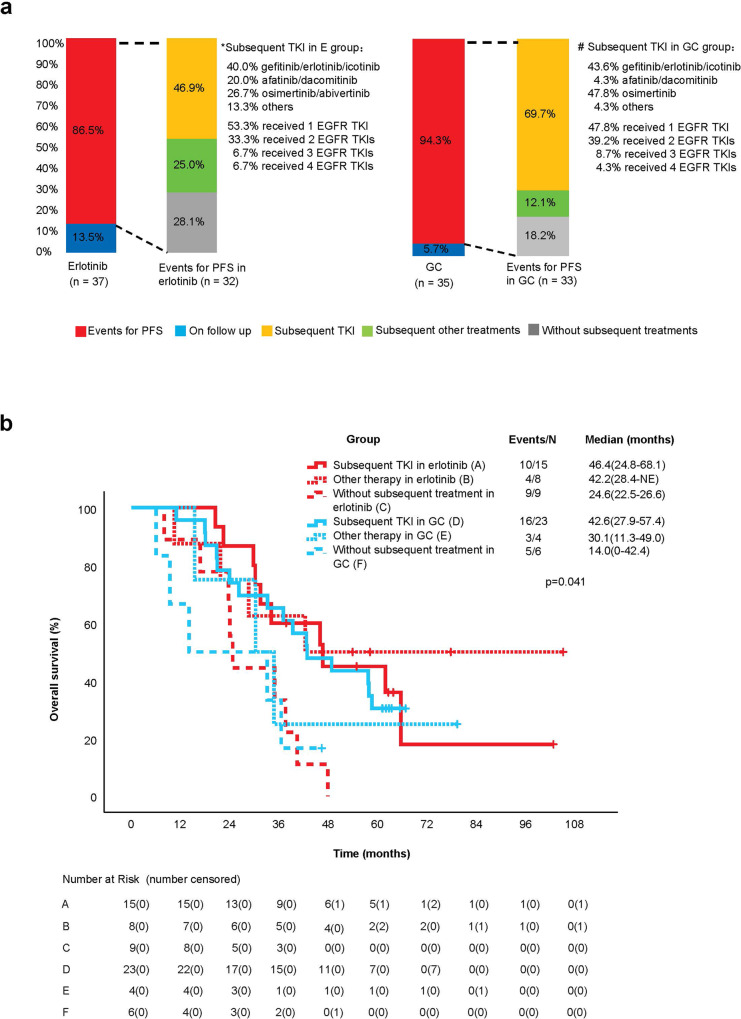
Subsequent treatments in **a** intention-to-treat population and **b** overall survival for subsequent treatments. E erlotinib, GC gemcitabine plus cisplatin, EGFR epidermal growth factor receptor, TKI tyrosine kinase inhibitor, PFS progression-free survival, NC not calculable

There were 65 (90.3%) patients in the ITT population who had disease relapse or death at the data cut-off. In patients experiencing a relapse, subsequent treatment was administered to 71.9% (23/32) in the erlotinib group and 81.8% (27/33) in the GC group (Table 3). In the erlotinib group, 46.9% of patients (15/32) received targeted therapy alone or combined with chemotherapy/local treatment, and 25.0% (8/32) received other treatments (chemotherapy with or without local treatment). In the GC group, 69.7% of patients (23/33) received targeted therapy alone or combined with chemotherapy/local treatment, and 12.1% (4/33) received other treatments (Fig. 3a). The proportion of subsequent treatments between the two groups was nonsignificant (*p* = 0.165).

**Table 3:** Subsequent treatments after disease relapse

Group	Erlotinib group (*n* = 32)	GC group (*n* = 33)	Total (*n* = 65)
With subsequent treatments	23 (71.9)	27 (81.8)	50 (76.9)
Targeted therapy	15 (46.9)	23 (69.7)	38 (58.5)
Other treatments	8 (25.0)	4 (12.1)	12 (18.4)
Without subsequent treatments	9 (28.1)	6 (18.2)	15 (23.1)
Total, *n* (%)	32 (100.0)	33 (100.0)	65 (100.0)

Data are *n* (%)

*GC* gemcitabine plus cisplatin

The most common site of metastases for the patients who had disease relapse was the lung (9,33.3%), lymph nodes (7,25.9%), bone (7,25.9%) and brain (6,22.2%) in the erlotinib group, the lung (18,58.1%), brain (6,19.4%), and lymph nodes (4,12.9%) metastases were most frequent in the GC group (Supplementary Table 1).

In the erlotinib group, the median OS with subsequent targeted therapy was 46.4 months (95% CI, 24.8–68.1) (group A), 42.2 months (95% CI, 28.4–not evaluable (NE)) with other subsequent treatments (group B), and 24.6 months (95% CI, 22.5–26.6) for the patients not receiving any subsequent treatment (group C). In the GC group, the median OS was 42.6 months (95% CI, 27.9–57.4) with subsequent targeted therapy (group D), 30.1 months (95% CI, 11.3–49.0) with other treatments (group E), and 14.0 months (95% CI, 0.0–42.4) for the patients not receiving any subsequent treatment (group F) (Fig. 3b).

For the patients with subsequent targeted therapy, the ORR was 53.3% (8/15) and the disease control rate (DCR) was 93.3% (14/15) in the erlotinib group, and 47.8% (11/23) and 73.9% (17/23), respectively, in the GC group. In addition, the ORR for patients with subsequent osimertinib was 25.0% (1/4), and 45.5% (5/11) for those without subsequent osimertinib in the erlotinib group.

The median duration of neoadjuvant erlotinib was 42 days (range, 20–48). A dose adjustment was required in one patient (2.7%) in the erlotinib group owing to the onset of adverse events. In the GC group, one patient (2.9%) refused chemotherapy and discontinued the study before initiating treatment. Two patients (5.7%) received one cycle, and 32 patients (91.4%) received two cycles of neoadjuvant GC treatment; six (17.1%) patients required dose adjustments for adverse events (Supplementary Table 2).

The median post-progression survival (PPS) was 19.6 months (95% CI, 8.1–31.2) in the erlotinib group and 27.6 months (95% CI, 7.0–48.3) in the GC group. The PPS was nonsignificant between the two groups (HR, 1.07; 95% CI, 0.60–1.91; *p* = 0.806) (Supplementary Fig. 1).

### Safety

The population for safety comprised 37 patients who received erlotinib and 34 who received GC except for one patient who refused to receive chemotherapy after randomisation. The adverse events (AEs) of any grade occurred in 70.3% (26/37) of patients with erlotinib and 58.8% (20/34) with GC during postoperative therapy. In brief, the most common AEs were rash (43.2%), diarrhoea (24.3%) and cough (24.3%) in patients treated with erlotinib; and those in the GC group were neutropenia (38.2%), decreased white blood cell count (32.4%), anorexia (26.5%) and vomiting (26.5%). The grade 3 or 4 AEs occurred in 5 (13.5%) patients of the erlotinib group and in 10 (29.4%) patients of the GC group. The most common grade 3 or 4 AEs were rash (5.4%), diarrhoea (2.7%), shortness of breath (2.7%), elevated total bilirubin (2.7%), elevated aminotransferases (2.7%), and decreased white blood cell count (2.7%) in the erlotinib group. In the GC group, those were neutropoenia (29.4%), decreased white blood cell count (11.8%), vomiting (2.9%), nausea (2.9%), anaemia (2.9%) and dyspnoea (2.9%).

## Discussion

IIIA-N2 NSCLC with potentially resectable disease (IIIA3 with N2 confirmed by EBUS/Mediastinoscopy or PET/CT) represents a highly heterogeneous disease in treatment modalities and prognosis. The EMERGING-CTONG 1103 study is the first randomised phase II trial to evaluate the feasibility and safety of neoadjuvant and adjuvant targeted therapy with erlotinib compared with standard chemotherapy. The results of PFS have previously been published in the *Journal of Clinical Oncology*.^17^ Herein, we report the final OS data of this study and found that the median OS of neoadjuvant erlotinib was 42.2 months, which is a promising result for patients with completely resected IIIA-N2 (IIIA3) NSCLC. The subgroups analysis for OS between the erlotinib group and the GC group exhibited that the OS benefit across all subgroups, including age, gender, N2 status, or *EGFR* mutation type. Despite no OS benefit, perioperative erlotinib continues to show superior PFS compared with GC.

The CHEST study showed that preoperative Cisplatin and Gemcitabine followed by radical surgery had significantly prolonged PFS and OS compared with surgery alone in patients with clinical stage IIB/IIIA NSCLC.^18^ Clinically, stage IIIA-N2 NSCLC represents a highly heterogeneous disease,^19^ making it challenging to select the most appropriate treatment. Despite multiple treatment modalities, the prognosis of these patients remains unsatisfactory, and survival times are highly variable. Previous randomised trials and meta-analyses have suggested that neoadjuvant/adjuvant chemotherapy could improve OS.^20,21^ However, only a small group of patients may benefit from such highly toxic treatment. Furthermore, prognostic benefits are limited regardless of surgery and adjuvant therapy, particularly in patients with stage IIIA NSCLC.

The CTONG 1104 study was the first to introduce targeted therapy into the post-operative setting and showed remarkable improvements in disease-free survival (DFS) compared with conventional chemotherapy.^22^ The phase II EVAN study^23^ and our EMERGING study (CTONG 1103)^17^ yielded breakthrough results in DFS or PFS with perioperative targeted therapy for patients with driver gene-positive stage IIIA NSCLC. Thus, these results support the expanded use of targeted therapy in neoadjuvant and adjuvant therapies to treat one of the most heterogeneous diseases, stage IIIA-N2 NSCLC. The current analysis continues to emphasise the substantial role of targeted therapy in the perioperative setting. Although all enroled patients were radiologically or pathologically diagnosed with N2 disease, the 5-year OS rate was 40.8%, which is an improvement over the historical stage IIIA NSCLC data of 23 and 38% for clinical N2 and R0 resections, respectively.^24^

In addition, these updated results continued to demonstrate superior median PFS with erlotinib compared with chemotherapy, with approximately 10 months of PFS benefit. However, this PFS benefit did not translate into a significant difference in OS between the erlotinib and GC groups. This finding may result from the complex multifactorial therapeutic approaches and the introduction of other highly potent EGFR-TKIs during later lines of treatment. Related to this is the fact that more patients in the GC group received EGFR-TKIs than those in the erlotinib group (69.7% vs. 46.9%) when disease progression occurred; such therapeutic crossover during subsequent treatment may have confounded the OS results. Unsurprisingly, patients without subsequent treatment had the worst prognosis in both groups.

We also explored whether patients who received upfront EGFR-TKIs may retain sensitivity to subsequent EGFR-TKIs and achieve a survival benefit. Among patients receiving subsequent EGFR-TKIs after disease progression, ORRs were 53.3% (erlotinib) and 47.8% (GC), and respective disease control rates were 93.3% (erlotinib) and 73.9% (GC). However, only 25% of patients in the erlotinib group responded to subsequent osimertinib treatment, while the ORR was 45.5% for patients who did not receive subsequent osimertinib. Due to the small number of patients receiving osimertinib (*n* = 4) in the erlotinib group, these data must be interpreted with caution and we need to study accordingly in more patients. In addition, there was no data were observed that patients received subsequent immunotherapy after disease progression. Several previous studies have shown that the response rate is low with immunotherapy after disease progression in NSCLC with EGFR mutation.^25–27^ So the use of immunotherapy in these patients remains controversial.

Recent advances in EGFR-TKI therapy development have resulted in highly potent EGFR-TKIs with increased intracranial penetration that might be additional therapeutic options for this patient population after disease progression. The ADAURA study,^28^ which was the first to investigate a third-generation EGFR-TKI in the adjuvant setting, showed remarkable preliminary DFS improvement in patients with stage IB-IIIA *EGFR*-mutant NSCLC (HR [osimertinib vs. placebo], 0.20; 95% CI, 0.14–0.30; *p* < 0.001).^29^ Given that our previous analysis of the CTONG 1104 study^22^ indicated a unique spatial–temporal treatment failure pattern with increased intracranial metastasis, it is possible that postoperative osimertinib could significantly lower the incidence of intracranial lesions, resulting in DFS improvement. In light of these findings, the randomised NeoADAURA trial (NCT04351555) was initiated, with the aim of determining whether perioperative osimertinib could further improve the prognosis for these patients. Biomarker analysis from CTONG 1104 showed that patients harbouring different co-mutations or T cell receptors would influence overall survival.^30,31^ Collectively, these data support the use of perioperative targeted therapy, instead of chemotherapy, as the preferred treatment option for patients with resectable stage IIIA-N2 *EGFR*-mutant NSCLC.

One of the main limitations of this study was that not all enroled patients had pathologically confirmed N2 disease, which may have led to an underestimation of the disease stage, thereby influencing the robustness of the survival analysis. Another limitation is that we did not obtain biopsy samples of all the recurrent lesions and could not further investigate if upfront targeted therapy may biologically impact subsequent treatment. Then, RCT studies with a larger sample size are needed to further explore the benefit of neoadjuvant EGFR-TKI on stage IIIA-N2 EGFR-mutant patients with NSCLC in the future.

In conclusion, the updated analysis of CTONG1103 indicated that erlotinib continued to improve PFS and OS numerically compared with platinum-based chemotherapy. Moreover, there was no evidence of cumulative toxicity in erlotinib during the long-term follow-up. The present results support the use of erlotinib in both the neoadjuvant and adjuvant settings for resectable stage IIIA-N2 *EGFR*-mutant NSCLC.

## Materials and methods

### Ethics statements

This study was approved by the Ethics Committee of Guangdong Provincial People’s Hospital (No. [2011] 28, Full names of the Ethics committees are: Jinrui Ou, Jianxing Cui, Nianqiao Zhang, Jianwei Mo, Deying Qian, Jimei Chen, Feizhou Jiang, Zuoyue Liu, Peihua Zheng, You Huang). All patients provided written informed consent prior to participating in the study.

### Study design

The EMERGING-CTONG 1103 study was a multicentre (17 centres in China), national, open-label, phase II, randomised controlled trial for comparing erlotinib with GC as neoadjuvant/adjuvant therapy in patients with stage IIIA-N2 NSCLC and exon 19 or 21 *EGFR* mutations. *EGFR* mutation status detection will be performed in the central laboratory by using a quantitative polymerase chain reaction (ADx-ARMS kit; Amoy Diagnostics, Xiamen, China). Full details of the study design have been published.^17^

### Patients

As previously described,^17^ patients eligible for the study had untreated, potentially resectable stage IIIA-N2 NSCLC with sensitive *EGFR* mutations, an Eastern Cooperative Oncology Group performance status of 0–1, a life expectancy of 12 weeks or more, and adequate organ function. Exclusion criteria included poor lung function, a history of malignancies, and historical/current interstitial lung disease.

### Randomisation and masking

All patients were randomly assigned in a 1:1 ratio to receive either of the two interventions by computer. Treatments were randomly assigned based on single-station N2 or multiple-station N2, adenocarcinoma or non-adenocarcinoma, never smoked or former smoked or currently smoked, male or female. Neither the study investigators nor the patients were masked.^17^

### Treatment

One group received neoadjuvant therapy with erlotinib 150 mg/day orally for 42 days and adjuvant therapy with erlotinib 150 mg/day orally for up to 12 months. The Chemo group received neoadjuvant therapy with gemcitabine 1250 mg/m^2^ plus cisplatin 75 mg/m^2^ intravenously for two cycles and adjuvant therapy with GC for up to two cycles.

### Outcomes

Details of dynamic assessment were described previously.^17^ The primary endpoint of the study was ORR, which was defined as the percentage of patients with a confirmed complete or partial response based on the Response Evaluation Criteria in Solid Tumours criteria version 1.1.

Secondary endpoints included: (1) lymph node downgrade rate defined as the proportion of patients with pathological confirmed lymph nodes downstaging from N2 to N1 or N0 in the intention to treat (ITT) population. (2) complete resection rate defined as the proportion of patients who received complete resection (R0 section) in the intention to treat (ITT) population. (3) pCR rate is determined as % residual viable tumour cells in the primary tumour and sampled lymph nodes. (4) OS was defined as the time from random assignment to the date of death from any cause, or data on patients were censored at the last confirmation of their survival. OS at 3 and 5 years is defined as the percentage of people still alive 3 or 5 years after the day of randomisation. (5) PFS defined as the time from surgery to the first confirmed disease progression or death from any cause, or data on patients were censored at the last tumour assessment. (6) safety (assessed by the US National Cancer Institute Common Terminology Criteria for Adverse Events version 4.0).

### Statistical analysis

Details of sample size calculations were described previously.^17^ Efficacy was assessed in the intention-to-treat population, which was defined as all randomised subjects. Safety was assessed in the safety population, which included all randomised subjects who received at least one dose of study treatment.

An Independent Review Committee (IRC) provided a review of the patient’s images, including CT, MRI, PET/CT and bone scan. Differences in the OS, PFS and the cumulative proportion of patients surviving at 3 and 5 years were compared using the Kaplan–Meier method. The response rate between the subsequent treatments was assessed using the Chi-square test. The effect of neoadjuvant treatment on OS in predefined subgroups (age, gender, N2 status and *EGFR* mutation) was assessed using Cox proportional hazard models presented in a forest plot.

Based on the investigator’s evaluation of the tumour response from patients’ medical records, Post hoc analyses for subsequent treatments were conducted for patients who experienced relapse or progression after surgery. All analyses were performed using SPSS 25.0 (IBM, Armonk, NY, USA) and R statistical packages (3.4.3). All tests were two-sided and *p* < 0.05 was considered statistically significant. The data cut-off date was 29 January 2021.

## Supplementary information

Supplementary materials Clean

## Data Availability

Overall clinicopathological data were summarised in corresponding tables. All other relevant individual data are available from the corresponding author of this study (Y.-L.W., syylwu@live.cn) upon reasonable request.
