# Supplementary material for: Erlotinib versus gemcitabine plus cisplatin as neoadjuvant treatment of stage IIIA-N2 EGFR-mutant non-small-cell lung cancer: final overall survival analysis of the EMERGING-CTONG 1103 randomised phase II trial
Source: Signal Transduct Target Ther. 2023 Feb 24;8:76. doi: 10.1038/s41392-022-01286-3 (PMC9950485; doi:10.1038/s41392-022-01286-3)
Supplement: Supplementary file 1 — Supplementary materials Clean [file 41392_2022_1286_MOESM1_ESM.doc]

Supplementary Materials for

**Erlotinib versus gemcitabine plus cisplatin as neoadjuvant treatment of stage IIIA-N2 *EGFR*-mutant non-small-cell lung cancer: final overall survival analysis of the EMERGING-CTONG 1103 randomised phase II trial**

Wen-Zhao Zhong^1^, Hong-Hong Yan^1^, Ke-Neng Chen^2^, Chun Chen^3^, Chun-Dong Gu^4^, Jun Wang^5^, Xue-Ning Yang^1^, Wei-Min Mao^6^, Qun Wang^7^, Gui-Bin Qiao^1,8^, Ying Cheng^9^, Lin Xu^10^, Chang-Li Wang^11^, Ming-Wei Chen^12^, Xiao-Zheng Kang^2^, Wan-Pu Yan^2^, Ri-Qiang Liao^1^, Jin-Ji Yang^1^, Xu-Chao Zhang^1^, Si-Yang Liu^1^, Qing Zhou^1^, Yi-Long Wu^1*^

Correspondence to: syylwu@live.cn

**This PDF file includes:**

Materials and Methods

Figures. S1

Tables S1 to S2

**Other Supplementary Materials for this manuscript include the following:**

Final protocol

**Materials and Methods**

Ethics statements

This study was approved by the Ethics Committee of Guangdong Provincial People’s Hospital (No. [2011] 28,Full names of the Ethics committees are: Jinrui Ou, Jianxing Cui, Nianqiao Zhang, Jianwei Mo, Deying Qian, Jimei Chen, Feizhou Jiang, Zuoyue Liu, Peihua Zheng, You Huang). All patients provided written informed consent prior to participating in the study.

Study design

The EMERGING-CTONG 1103 study was a multicentre (17 centres in China), national, open-label, phase II, randomised controlled trial for comparing erlotinib with GC as neoadjuvant/adjuvant therapy in patients with stage IIIA-N2 NSCLC and exon 19 or 21 EGFR mutations. EGFR mutation status detection will be performed in the central laboratory by using quantitative polymerase chain reaction (ADx-ARMS kit; Amoy Diagnostics, Xiamen, China). Full details of the study design have been published 17.

Patients

As previously described 17, patients eligible for the study had untreated, potentially resectable stage IIIA-N2 NSCLC with sensitive EGFR mutations, an Eastern Cooperative Oncology Group performance status of 0 to 1, a life expectancy of 12 weeks or more, and adequate organ function. Exclusion criteria included poor lung function, a history of malignancies, and historical/current interstitial lung disease.

Randomisation and masking

All patients were randomly assigned in a 1:1 ratio to receive either of the two interventions by computer. Treatments were randomly assigned based on single-station N2 or multiple station N2, adenocarcinoma or non-adenocarcinoma, never smoked or former smoked or currently smoked ,male or female. Neither the study investigators nor the patients were masked 17.

Treatment

One group received neoadjuvant therapy with erlotinib 150 mg/day orally for 42 days and adjuvant therapy with erlotinib 150 mg/day orally for up to 12 months. The Chemo group received neoadjuvant therapy with gemcitabine 1250 mg/m2 plus cisplatin 75 mg/m2 intravenously for two cycles and adjuvant therapy with GC for up to two cycles.

Outcomes

Details of dynamic assessment were described previously 17.The primary endpoint of the study was ORR, which was defined as the percentage of patients with a confirmed complete or partial response based on the Response Evaluation Criteria in Solid Tumors criteria version 1.1.

Secondary endpoints included: (1) lymph node downgrade rate defined as the proportion of patients with pathological confirmed lymph nodes downstaging from N2 to N1 or N0 in the intention to treat (ITT) population. (2) complete resection rate defined as the proportion of patients who received completely resection (R0 section) in the intention to treat (ITT) population. (3) pCR rate is determined as % residual viable tumor cells in the primary tumor and sampled lymph nodes. (4) OS was defined as the time from random assignment to the date of death from any cause,or data on patients were censored at the last confirmation of their surviva. OS at 3 and 5 years is defined as the percentage of people still alive following 3 or 5 years after the day of randomization.(5) PFS defined as the time from surgery to the first confirmed disease progression or death from any cause, or data on patients were censored at the last tumor assessment.(6) safety (assessed by the US National Cancer Institute Common Terminology Criteria for Adverse Events version 4.0).

Statistical analysis

Details of sample size calculations were described previously 17. Efficacy was assessed in the intention-to-treat population, which was defined as all randomised subjects. Safety was assessed in the safety population, which included all randomised subjects who received at least one dose of study treatment.

An independent review committee (IRC) provided review for the patient's images, including CT, MRI, PET/CT and bone scan. Differences in the OS, PFS , and the cumulative proportion of patients surviving at 3 and 5 years were compared using Kaplan-Meier method. The response rate between the subsequent treatments were assessed using the Chi-square test. The effect of neoadjuvant treatment on OS in predefined subgroups (age, gender, N2 status and EGFR mutation) was assessed using Cox proportional hazard models presented in a forest plot.

Based on investigator evaluation of the tumour response from patients’ medical records, Post hoc analyses for subsequent treatments were conducted for patients who experienced relapse or progression after surgery. All analyses were performed using SPSS 25.0 (IBM, Armonk, NY, USA) and R statisitcal packages (3.4.3). All tests were two sided and p< 0.05 was considered statistically significant. The data cut-off date was 29 January 2021.

Data Availability

Overall clinicopathological data were summarised in corresponding tables. All other relevant individual data are available from the corresponding author of this study (Yi-Long Wu, syylwu@live.cn) upon reasonable request.

**Figure. S1.**

**Group Events/N Median (months,95% CI)**

Erlotinib 23/37 19.6 (8.1-31.2)

100

GC 24/35 27.6 (7.0-48.3)

Post-progression survival (%)

Hazard ratio (95% CI) = 1.07(0.60-1.91)

*p* = 0.806

80

60

40

20

0

0 12 24 36 48 60 72 84 96

Number at Risk (number censored)

Erlotinib 37(0) 23(1) 15(2) 7(4) 4(3) 2(2) 1(1) 1(0) 0(1)

GC 35(0) 23(1) 19(0) 12(2) 7(2) 2(4) 1(1) 0(1) 0(0)

**Supplementary Figure S1.** Kaplan–Meier analysis of post-progression survival in the intention-to-treat population.

Table S1. **The failure patterns for the patients had relapsed***

| The failure patterns | Erlotinib group | GC group |
| --- | --- | --- |
|  | (n=27) | (n=31) |
| lung | 9 (33.3%) | 18 (58.1%) |
| lymph nodes | 7 (25.9%) | 4 (12.9%) |
| bone | 7 (25.9%) | 3 (9.7%) |
| brain | 6 (22.2%) | 6 (19.4%) |
| pleura | 1 (3.7%) | 3 (9.7%) |
| liver | 1 (3.7%) | 1 (3.2%) |
| para-abdominal aorta | 1 (3.7%) | 0 (0) |
| Pericardial effusion | 1 (3.7%) | 0 (0) |
| pancreatic tail | 1 (3.7%) | 0 (0) |
| pleural effusion | 0 (0) | 1 (3.2%) |
| kidney | 0 (0) | 1 (3.2%) |

Data are n (%) unless otherwise stated. *There were 58 relapsed and 7 deaths before relapsed across 65 progression events.GC=gemcitabine plus cisplatin.

Table S2. Summary of neoadjuvant treatment (intention-to-treat population)

|  | Erlotinib group | GC group |
| --- | --- | --- |
|  | (n=37) | (n=35)* |
| Treatment duration, days |  |  |
| Median (range) | 42 (20–48) | — |
| Cycles |  |  |
| One cycle | — | 2 (5.7) |
| Two cycles | — | 32 (91.4) |
| Dose adjustment | 1 (2.7) | 6 (17.1) |
| Dose adjustment due to adverse event | 1 (2.7) | 6 (17.1) |

Data are n (%) unless otherwise stated. *One patient in the GC group discontinued before receiving treatment. GC=gemcitabine plus cisplatin.

**Final protocol**

**Erlotinib versus gemcitabine plus cisplatin as neoadjuvant treatment for stage IIIA–N2 non-small-cell lung cancer (NSCLC) with EGFR mutation in exon 19 or 21: a multicenter, randomized controlled trial**

**Protocol ID number：ML 25304, C-TONG 1103**

**Sponsor: Guangdong Association of Clinical Trials**

Declaration of Secrecy

The information contained in this document (especially unpublished data) belongs to the sponsor. Hence, the information provided to you (investigator, potential investigator or consultant) is confidential and can only be used by you, your employees or relevant ethics committee’s review. The information in this document should not be disclosed to others without the written permission of the sponsor, unless in cases where information is required to obtain informed consent from a patient who may take the drug.

**Summary of study protocol**

| **Study title** | Erlotinib versus gemcitabine plus cisplatin as neoadjuvant treatment for stage IIIA–N2 non-small-cell lung cancer (NSCLC) with EGFR mutation in exon 19 or 21: a multicenter, randomized controlled trial | | |
| --- | --- | --- | --- |
| **Protocol ID number/Version/Date** | ML 25304, C-TONG 1103 / Version 3.0/ Nov 24^th^, 2017 | | |
| **Sponsor** | Guangdong Association of Clinical Trials | Tel | 020-83827812-51221 |
| **Indication** | Previously untreated resectable stage IIIA–N2 non-small-cell lung cancer (NSCLC) with epidermal growth factor receptor (EGFR) mutation in exon 19 or 21 | | |
| **Objectives** | **Primary：**  To investigate the objective response rate (ORR) of erlotinib versus gemcitabine plus cisplatin as neoadjuvant treatment for stage IIIA–N2 NSCLC with EGFR mutation in exon 19 or 21. | | |
|  | **Secondary：**   - To compare downstaging rates of pathological lymph nodes of two study groups after neoadjuvant treatment； - To compare complete resection rate of two study groups； - To compare pathological complete response (pCR) rate of two study groups; - To compare progression-free survival (PFS) of two study groups; - To compare 3-year and 5-year survival rate of two study groups; - To investigate safety of two study groups using NCI CTC-AE (Version 4.0)； - To compare quality of life (QoL) of two study groups.   **Exploratory：**  Post-operative 24 week, 48 week and 3 year DFS rate，exploratory biomarker analysis (the intending biomarkers includes T790M, cMET, KRAS, ALK-EML4, BRAF, PTEN). | | |
| **Study design** | Prospective, open-label, randomized controlled, multicenter, phase II study | | |
| **Patient number** | 72 | | |
| **Target population** | Study population includes previously untreated resectable stage IIIA–N2 NSCLC patients, and biopsy tissue from primary lesion or metastatic lymph node comfirmed patients with EGFR mutation in exon 19 or 21. | | |
| **Inclusion Criteria** | **Inclusion criteria related to disease:**   - Histologically or cytologically confirmed NSCLC; - Previously untreated with operation, chemotherapy, biotherapy and radiotherapy; - Resectable stage IIIA–N2 NSCLC patients by investagotor assessment, and biopsy tissue from primary lesion or metastatic lymph node comfirmed patients with EGFR mutation in exon 19 or 21. Patients had N2 disease confirmed by mediastinoscopy, endobronchial ultrasonography (EBUS), PET/CT or thoracoscope. - Sufficient tumor tissue (not cytology specimens) available for molecular marker analyses; - At least one measurable lesion with a largest diameter of ≥10 mm on spiral CT according to Response Evaluation Criteria in Solid Tumors version 1.1 (RECIST 1.1).   **Hematology, biochemistry and organ function:**   - Pulmonary ventilation function, FEV1≥1.5L or anticipated FEV1≥800ml after lobectomy or pneumonectomy; - Hemoglobin ≥ 9.0 g/dL (can be maintained or exceed by blood transufusion); - Absolute neutrophil count ≥1.5×10^9^/L； - Blood platelet count ≥100×10^9^/L； - Total bilirubin ≤1.5 times the upper limit of normal; - AST and ALT ≤2.5 times the upper limit of normal; - Creatinine ≤1.25 times the upper limit of normal; and creatinine clearance rate ≥60mLl/min; - International normalized ration (INR) of prothrombin time in patients without anticoagulant therapy ≤1.5, partial thromboplastin time (APTT) ≤1.5 times the upper limit of normal. Patients treated with full dose or parenteral anti-coagulation treatment were eligible if the dose of anticoagulant was stable for at least 2 weeks before enrolling the clinical trial and the result of blood conagulation test belonged to the range defined by the local laboratory； - Child-bearing women (15-49 years) should have urine pregnancy test within 7 days before treatment, and the result should be negative.   **General inclusion criteria:**   - Provision of signed informed consent by patients or their legal representative; - Patients received oral drugs according to study protocol and follow-up process; - Patients aged ≥18 years old; - Patients can tolerate neoadjuvant therapy (including neoadjuvant chemotherapy) and operation, ECOG performance status 0-1, life expectarncy >12 weeks; - Men and women of childbearing age agreed to adopt a reliable method of contraception before enrolling the trial, during the research and within 30 days after stopping the drug. | | |
| **Exclusion criteria** | - Any previous systemic anti-cancer cancer therapy for NSCLC, including cytotoxic therapy, target therapy (including tyrosine kinase inhibitor or monoclonal antibody) and any other investigational agent ; - Pevious local radiotherapy for NSCLC; - Patients with preious malignancies other than NSCLC within 5 years, except for cured in situ carcinoma of the uterine cervix, cured basal cell carcinoma and bladder epithelial tumor [including Ta and Tis]; - Any unstable systemic disease, including active infection, uncontrolled hypertension, unstable angina, angina that began to attack in the last 3 months, congestive heart failure (≥ New York Heart Association [NYHA] class II), myocardium infarction (6 months befor enrollment), severe arrhythmia requiring medication, liver, kidney or metabolic disease； - Previously or currently with interstitial lung disease; - Incomplete or uncontrolled eye inflammation or eye infection, or any condition that may cause the above mentioned eye diseases; - Confirmed human immunodeficiency virus (HIV) infection; - Allergic to erlotinib or gemcitabine or cisplation; - Patients who underwent major operation or severe trauma within 2 months prior to the first dose; - Mixed with small cell lung cancer; - Pregnant or lactating women; - Investigartors evulate that patient was not suitable for enrollment, such as neurological or metabolic disorders, physical examination or laboratory suspecting that patients have a possible disease or have contraindications to study drug use, or has a high risk of treatment-related complications. | | |
| **Study period** | Each enrolled patient had a neoadjuvant treatment period for approximately 2 months, operation and post-operative recovery period for about 1 months, follow-up for 2 years after operation and then survival follow-up for 3 years. In total, the longest study time for every enrolled patient was about 64 months.  Calculated according to approximately 5 patients per month, this study intended to enroll 72 patients, and it would take 18 months for enrollment. Patients will be follow-up for 2 years after operation and then had survival follow-up for 3 years. The total study period takes approximately 82 months. | | |
| **End of study** | The study ended with the first occurrence of the following: the last enrolled patients had received operation for 5 years or had disease progression during neoadjuvant treatment for 5 years. | | |
| **Dose of study drug /administration route/program** | **Patients randomized to erlotinib or chemotherapy group**  **Neoadjuvant therapy**  **Erlotinib group:** erlotinib 150 mg/day administered orally for 6 weeks (42 days), treated until disease progression or unacceptable toxicity.  **Chemotherapy group:** gemcitabine 1250mg/m^2^ administered intravenously on day 1 and day 8.  Cisplatin 75 mg/m2 administered intravenously on day 1.  Every 3 weeks for 2 cycles, treated until disease progression or unacceptable toxicity.  **Post-opearation**  **Erlotinib group:** If investigators considered post-operative adjuvant thearapy, then patients would receive erlotinib for 12 months after surgery or until disease progression or unacceptable toxicity. Erlotinib 150 mg/day administered orally.  **Chemotherapy group:** If investigators considered post-operative adjuvant thearapy, then patients would receive chemotherapy for 2 cycles (every 3 weeks for 1 cycle) after surgery or until disease progression or unacceptable toxicity.。  Gemcitabine 1250mg/m^2^ administered intravenously on day 1 and day 8.  Cisplatin 75 mg/m^2^ administered intravenously on day 1. | | |
| **Study endpoint** | **Primary endpoint:**   - Objective response rate (ORR).   **Sencondary endopoint:**   - Downsating rates of pathological lymph nodes; - Complete resection rate； - Pathological complete response (pCR) rate； - Progression-free survival (PFS); - 3-year and 5-year OS rate; - Quality of life (QoL); - Safety.   **Exploratory analysis：**   - Post-operative 24 week, 48 week and 3 year disease free survival (DFS) rate; - Biomarker analysis (potentinal biomarkers, T790M, cMET, KRAS, ALK-EML4, BRAF, PTEN). | | |
| **Safety** | All patients who received at least one dose of study drug will be included in the safety population. Patients’ physical examination results, vital signs, adverse events, and laboratory test abnormality of will be summarized. Adverse event will be reported and graded in terms of NCI Common Terminology Criteria for Adverse Events (CTCAE) version 4.0. | | |
| **Quality of life (QoL)** | QoL will be evaluated by Functional Assessment of Cancer Therapy-Lung questionnaire (FACT-L) version 4 and Lung Cancer Symptom Scale (LCSS). | | |

# Summary of study design

Erlotinib 150mg/d

6 weeks

Surgery

Erlotinib 150mg/d

1 year

Stage IIIA-N2 NSCLC with EGFR sensitive mutation

Surgery

Gemcitabine 1250mg/m^2^ + Cisplatin 75mg/m^2^

Every 3 weeks for 1 cycle, 2 cycles

Gemcitabine 1250mg/m^2^ + Cisplatin 75mg/m^2^

Every 3 weeks for 1 cycle, 2 cycles

cycles

**Schedule of Activities（1）**

|  | **Screening** | **Baseline** | **Pre-operation phase ^(G)^** | | | | | | **Surgical evalution and surgery ^(J)^** | **Post-operation adjuvant therapy^(O)^** | **Recurrence visit follow-up ^(L)^** | **Survival follow-up** ^(M)^ |
| --- | --- | --- | --- | --- | --- | --- | --- | --- | --- | --- | --- | --- |
| **Cycle/Visit** |  |  | Cycle 1（d1-21） | | | Cycle 2（d22-42） | | |  |  |  |  |
| **Days** | d-28~d-7 | d-7~d-1 | d1 | d8 | d15 | d1 | d8 | d15 |  |  | Every 3 month | Every 3 month |
| Informed consent**^(A)^** | X | X |  |  |  |  |  |  |  |  |  |  |
| Inclusion/Exclusion criterion | X |  |  |  |  |  |  |  |  |  |  |  |
| Tumor tissue EGFR test **^(W)^** | X |  |  |  |  |  |  |  |  |  |  |  |
| Medical history (including smoking history) | X |  |  |  |  |  |  |  |  |  |  |  |
| Physical examination**^(B)^** | X |  | X |  |  | X |  |  | X |  |  |  |
| Vital signs, weight |  | X |  |  |  | X |  |  | X |  |  |  |
| ECOG PS |  | X |  |  |  | X |  |  | X |  |  |  |
| Pulmonary function | X |  |  | | | | | | | | | |
| ECG、Chest X-ray**^(F)^** | X |  | Acoording to clinical indications | | | | | | | | | |
| Abdominal enhanced CT | X |  |  |  |  |  |  |  | X | Acooording to clinical indications | | |
| Abdominal ultrasound |  |  |  |  |  |  |  |  |  |  | X**^(L)^** every 3 months |  |
| Bone scan | X |  |  |  |  |  |  |  |  |  | X**^(L)^** every 1 year |  |
| Cranial enhanced MRI**^(R)^** | X |  |  |  |  |  |  |  | X |  | X**^(L)^** every 6 months |  |
| Coagulation and HIV antibody **^(N)^** | X |  | Acoording to clinical indications | | | | | | | | | |
| Blood routine **^(C)^** |  | X |  | X**^(S)^** |  | X**^(Q)^** | X**^(S)^** |  | X | X **^(T)^** |  |  |
| Blood chemistry **^(C)^** |  | X |  |  |  | X**^(Q)^** |  |  | X | X **^(T)^** |  |  |
| Collecting blood samples for exploratory research **^(U)^** |  | X |  |  |  |  |  |  | X | X**^(U)^** | | |
| Urine routine **^(D)^** |  | X | Acoording to clinical indications | | | | | | | | | |
| Pregnancy test（If applicable）**^(E)^** |  | X | Acoording to clinical indications | | | | | | | | | |
| Accompanying disease and treatment | X |  | The entire treatment period and within 28 days after the last dose | | | | | | | | | |
| Randomization **^(K)^** |  | X |  |  |  |  |  |  |  |  |  |  |
| Gemcitabine (Chemotherapy group) |  |  | X | X |  | X | X |  |  | The same as pre-operation treatment |  |  |
| Cisplatin (Chemotherapy group) |  |  | X |  |  | X |  |  |  | The same as pre-operation treatment |  |  |
| Erlotinib **^(H)^** |  |  | X (d1~d42) | | | | | |  | Treatment for 1 year |  |  |
| Tumor assessment （RECIST）**^(Y)^** | X |  |  |  |  |  |  |  | X**^(I)^** | X**^(L)^** | X**^(L)^** |  |
| Surgery **^(I)^** |  |  |  |  |  |  |  |  | X |  |  |  |
| Collecting sugical tumor tissue **^(V)^** |  |  |  |  |  |  |  |  | X |  |  |  |
| Safety evaluation/ record |  |  | The entire treatment period and within 28 days after the last dose | | | | | | | | | |
| FACT-L/LCSS questionnaire **^(P)^** |  |  | X |  |  | X |  |  | X | X**^(P)^** |  |  |
| Post-opeartion follow-up and survival follow-up**^(M)^** |  |  |  |  |  |  |  |  |  |  | X | X |
| Collecting tumor tissue after tumor recurrence **^(X)^** |  |  |  |  |  |  |  |  |  |  |  |  |

During pre-operation phase, the first day window period of the second cycle is ±3 days; from post-operation follow-up to recurrence, the window period of every follow-up is ± 7 days; after recurrence, the window period of every follow-up is ±14 days.

（A）Written informed consent must be obtained prior to any screening procedures related to the trial. Before entering the screening period, patients must sign an informed conseent form for senting tissue specimen for EGFR testing. Patients must sign an informed consent form for participating the study prior to randomization.

（B）Including detailed system examination. Physical examination at screening includes height measurement.

（C）Before the start of each cycle of chemotherapy, investigators should obtain the results of corresponding laboratory tests and evaluate them according to the program. Patients in erlotinib group should retest blood routine and biochemical routine on the 2^nd^ day of neoadjuvant treatment.

（D）Urine routines must be examined during the screening period and subsequently examined according to clinical needs.

（E）Childbearing women need to have a urine pregnancy test (unless at least 12 months of amenorrhea). If pregnancy is suspected during the study, the urine pregnancy test should be repeated.

（F）If chest CT has been examined within 4 weeks prior to enrollment, X-ray examination is not necessary for screening.

（G）Every 21 days (3 weeks) was 1 cycle. Gemcitabine 1250 mg/m^2^ administered intravenously on day 1 and day 8 of each cycle；cisplation 75 mg/m^2^ administered intravenously on day 1 of each cycle.

（H）Erlotinib mg/day administered orally for 42 days.

（I）Response evaluation performed with 1 week (from 43 to 49 day) after 2 cycles of neo-adjuvant treatment. The investigator decided whether patient underwent surgery based on the results of the assessment.

（J）Surgery was performed within 2 weeks after neoadjuvant therapy (day 50 to 63). Surgical evaluation and surgical stage records included surgical records (resection range, number of tumors) and surgical pathology results.

（K）Randomization was on day -1. The window period of the first dosing after randomization was ±3 days.

（L）If patients received adjuvant treatment after surgery, a chest-enhanced CT examination should be performed within 28 days before the start of the adjuvant treatment. Postoperative follow-up visits were performed every 3 months from the first day of adjuvant therapy until disease recurrence. Thoracic-enhanced CT and abdominal B-ultrasound (including liver, gallbladder, spleen, pancreas, kidneys, and bilateral adrenal glands) were reviewed every 3 months. Cranial-enhanced MRI was reviewed every 6 months, and bone scan was reviewed every 12 months. The time, schedule, dose, and duration of postoperative chemotherapy and/or radiotherapy would be recorded. If patients did not receive adjuvant treatment after surgery, a chest-enhanced CT examination should be performed within 8 weeks. After that, patients would be follow-up every 3 months until disease recurrence. The follow-up included patients’ general condition, symptoms and signs. The chest-enhanced CT and abdominal B-ultrasound (including liver, gallbladder, spleen, pancreas, kidney, and bilateral adrenal glands) were reviewed every 3 months. The head-enhanced MRI was reviewed every 6 months. The bone scan was reviewed every 12 months. The time, schedule, dose, and duration of postoperative chemotherapy and/or radiotherapy would be recorded. Patients entered into survival follow-up after recurrence.

（M）Patients who recurred after surgery or in neoadjuvant therapy would be followed up every 3 months to 5 years after surgery or died.

（N）If warfarin or heparin must be used, coagulation should be closely monitored.

（O）Investigators at each center can decide whether to give postoperative adjuvant therapy to patients with stage IA or pathologic complete remission (pCR) based on specific conditions, but it is recommended to provide postoperative adjuvant therapy to all patients.

（P）During neoadjuvant treatment period, patients in the erlotinib and chemotherapy groups performed the FACT-L/LCSS questionnaire on day 1 of each cycle (namely day 1 and day 22) and day 43, and those questionnaires should be performed prior to drug administration on that day. During adjuvant treatment period, patients in the erlotinib and chemotherapy groups performed the FACT-L/LCSS questionnaire on day 1 of each cycle (namely day 1 and day 22) and day 43, and those questionnaires on day 1 and day 22 should be performed prior to drug administration on that day. After that, FACT-L/LCSS questionnaire would be performed at the time of every imaing revies until 1 year after adjuvant treatment (calculating from the 1^st^ day of adjuvant treatment), tumor recurrence, lost follow-up or death (which comes first).

（Q）Blood routine and blood biochemistry before the second cycle of treatment can be performed within 7 days before the first day of the second cycle.

（R）If patients have underdone PET/CT scan, they also need to have a cranical enhanced MRI.

（S）Patients in erlotinib group did not need to retest blood routine on the 8^th^ day of each cycle.

（T）The blood routine and biochemical routine examination procedures in the adjuvant treatment phase of the chemotherapy group were the same as the neoadjuvant treatment phase. After adjuvant therapy, patients did not need to undergo such tests; the blood routine and biochemical routine examination procedures in the first cycle (every 21 days for 1 cycle) of adjuvant treatment phase of the erlotinib group were the same as the neoadjuvant treatment phase; After adjuvant therapy, patients did not need to undergo such tests; after that, patients underwent the blood routine and biochemical routine examination every 3 months until the end of adjuvant therapy.

(U) Blood samples were collected for exploratory biomarker analysis. Time for blood collection: 1 sample before neoadjuvant therapy, 1 sample after neoadjuvant therapy, after that, blood sample collected as study follow-up within 2 years after sugerty; if patients recurred 2 years after surgery, then collecting blood sample at time of recurrence. The specific proceduere was show in the “SOP for collcetion of tissue and blood samples”.

（V）Part of the primary tumor and mediastinal lymph node obtained during the operation would be transferred and stored in fresh tissue and paraffin blocks (or paraffin sections). The specific proceduere was show in the “SOP for collcetion of tissue and blood samples”.

(W) During screening period, patients’ biopsy tissue from the primary tumor or mediastinal lymph node should be sent to centre laboratory for EGFR mutation testing. The specific proceduere was show in the “SOP for collcetion of tissue and blood samples”.

(X) When the tumor recurred after surgery, the biopsy tissue of recurrent lesion should be collected as much as possible. The specific proceduere was show in the “SOP for collcetion of tissue and blood samples”.

(Y) Tumor assessment refers to chest-enhanced CT scan, intends to evulate the exent of lung lesion. If PET/CT scan is used for tumor evaluation during screening period, the evaluation of efficacy after neoadjuvant therapy also requires PET/CT (PET/CT reassementment did not require abdominal enhanced CT at the same time). If patients recived adjuvant therapy after surgery, a chest enhanced CT scan should be performed within 28 days before the start of adjuvant therapy. Tumor assessment every 3 months from the first day of adjuvant therapy refers to chest enhanced CT scan; If patients did not recive adjuvant therapy after surgery, a chest enhanced CT scan was required within 8 weeks after surgery, then reassess chest-enhanced CT every 3 months.

**Contents**

Summary of study design [16](#__RefHeading___Toc408535802)

Abbreviation [17](#__RefHeading___Toc408535803)

Signature [19](#__RefHeading___Toc408535804)

1.1. Non-small cell lung cancer [20](#__RefHeading___Toc408535805)

1.2. Current status of incidience and treatment of stage ⅢA N2 non-small cell lung cancer [20](#__RefHeading___Toc408535806)

1.3. Background information of erlotinib [22](#__RefHeading___Toc408535807)

1.3.1 Second or third line erlotinib in advanced NSCLC [23](#__RefHeading___Toc408535808)

1.3.2 Erlotinib in EGFR mutant NSCLC [24](#__RefHeading___Toc408535809)

1.3.3 Rationale of dose selection of erlotinib [25](#__RefHeading___Toc408535810)

1.4 Rationale of study [25](#__RefHeading___Toc408535811)

1.4.2 The efficacy of EGFR-TKI and traditional platinum-containing chemotherapy in lung cancer patients with EGFR activating mutation [25](#__RefHeading___Toc408535812)

1.4.3 Potential benefit of pre-operative introduction target therapy [26](#__RefHeading___Toc408535813)

1.5 Questions to be addressed [26](#__RefHeading___Toc408535814)

2.1. Primary objective [26](#__RefHeading___Toc408535815)

2.2. Secondary objectives [27](#__RefHeading___Toc408535816)

2.3. Explortary analysis [27](#__RefHeading___Toc408535817)

3.1. Study design and planning [27](#__RefHeading___Toc408535818)

3.2 Randomization [28](#__RefHeading___Toc408535819)

3.3 Patients selection [28](#__RefHeading___Toc408535820)

3.3.1 Inclusion criteria [28](#__RefHeading___Toc408535821)

3.3.2 Exclusion criteria [29](#__RefHeading___Toc408535822)

3.4 Study period [30](#__RefHeading___Toc408535823)

3.5 Patients withdraw from study [30](#__RefHeading___Toc408535824)

3.6 Treatment plan [32](#__RefHeading___Toc408535825)

3.6.1 Treatment arrangement [32](#__RefHeading___Toc408535826)

3.6.2 Auxiliary medicaiton [34](#__RefHeading___Toc408535827)

3.6.3 Delay in drug delivery and dose adjustment of chemotherapy drugs [34](#__RefHeading___Toc408535828)

3.6.3 Delay in administration and dose adjustment of erlotinib [37](#__RefHeading___Toc408535829)

3.6.4 Combined treatment and smoking [37](#__RefHeading___Toc408535830)

3.6.5 Concomitant medication [38](#__RefHeading___Toc408535831)

3.6.6 Treatment compliance [38](#__RefHeading___Toc408535832)

3.7 Study endpints [38](#__RefHeading___Toc408535833)

3.7.1 Efficacy endpoint [39](#__RefHeading___Toc408535834)

3.7.2 Patients’ self-assessment [40](#__RefHeading___Toc408535835)

3.7.3 Safety parameters and definition [40](#__RefHeading___Toc408535836)

3.7.4 Evaluation period [40](#__RefHeading___Toc408535837)

3.8 Study process [41](#__RefHeading___Toc408535838)

3.8.1 Screening period and baseline [41](#__RefHeading___Toc408535839)

3.8.2 Neoadjuvant treatment period and surgery period [42](#__RefHeading___Toc408535840)

3.8.3 Post-operation adjuvant treatment period [43](#__RefHeading___Toc408535841)

3.8.4 Recurrence visit follow-up [43](#__RefHeading___Toc408535842)

3.8.5 Survival follow-up [44](#__RefHeading___Toc408535843)

3.8.6 Temporary visit [44](#__RefHeading___Toc408535844)

3.9 Quality of data [44](#__RefHeading___Toc408535845)

3.10 Archive [44](#__RefHeading___Toc408535846)

4 Ethical and condiserations [45](#__RefHeading___Toc408535847)

4.1 Independent ethics committee (IEC) [45](#__RefHeading___Toc408535848)

4.2 Ethical guidance for this study [45](#__RefHeading___Toc408535849)

4.3 Patient notice and informed consent [45](#__RefHeading___Toc408535850)

4.4 Confidentiality [45](#__RefHeading___Toc408535851)

4.5 Conditions for protocol amendment [46](#__RefHeading___Toc408535852)

4.6 Conditions for termination of study [46](#__RefHeading___Toc408535853)

4.7 Preservation of research documents, medical report forms and records [46](#__RefHeading___Toc408535854)

4.7.1 Preservation of investigators’ documents [46](#__RefHeading___Toc408535855)

4.7.2 Raw record and background data information [47](#__RefHeading___Toc408535856)

4.7.3 Audit and inspection [47](#__RefHeading___Toc408535857)

4.7.4 Case report form [47](#__RefHeading___Toc408535858)

5 Research audit [47](#__RefHeading___Toc408535859)

6 Publication of research data and protection of trade secrets [48](#__RefHeading___Toc408535860)

7 Statistics and analysis plan [48](#__RefHeading___Toc408535861)

7.1 Primary and secondary variables [48](#__RefHeading___Toc408535862)

7.1.1 Primary variable [48](#__RefHeading___Toc408535863)

7.1.2 Secondary variables [48](#__RefHeading___Toc408535864)

7.2 Statistics analysis [49](#__RefHeading___Toc408535865)

7.2.1 Baseline and demographic characteristics [49](#__RefHeading___Toc408535866)

7.2.2 Primary efficacy [49](#__RefHeading___Toc408535867)

7.2.3 Secondary efficacy [49](#__RefHeading___Toc408535868)

7.2.4 Exploratory analysis: [50](#__RefHeading___Toc408535869)

7.3 Hypothesis Testing [50](#__RefHeading___Toc408535870)

7.4 Analysis type [51](#__RefHeading___Toc408535871)

7.5 Interim analysis [51](#__RefHeading___Toc408535872)

7.6 Saftey analysis [51](#__RefHeading___Toc408535873)

7.7 Determination of sample size [51](#__RefHeading___Toc408535874)

8. Safety principle [52](#__RefHeading___Toc408535875)

8.1 Precautions/ warnings [52](#__RefHeading___Toc408535876)

8.1.1 Interstitial lung disease (ILD)-like adverse events [52](#__RefHeading___Toc408535877)

8.1.2 Diarrhea, dehydration, electrolyte imbalance and kidney failure [53](#__RefHeading___Toc408535878)

8.1.3 Hepatitis, liver failure [53](#__RefHeading___Toc408535879)

8.1.4 Gastrointestinal perforation [53](#__RefHeading___Toc408535880)

8.1.5 Bullous and exfoliative skin discorders [53](#__RefHeading___Toc408535881)

8.1.6 Ocular disorders [53](#__RefHeading___Toc408535882)

8.1.7 Toxicity caused by interaction between drugs [54](#__RefHeading___Toc408535883)

8.2 Adverse events [54](#__RefHeading___Toc408535884)

8.2.1 Definition of adverse events [54](#__RefHeading___Toc408535885)

8.2.2 Grade of adverse events [54](#__RefHeading___Toc408535886)

8.2.3 Monitoring of adverse events [55](#__RefHeading___Toc408535887)

8.2.4 Relationship between adverse events and study drug [55](#__RefHeading___Toc408535888)

8.2.5 Serious adverse event (SAE): [56](#__RefHeading___Toc408535889)

8.2.6 Non-serious adverse events of special interest (AESI) [57](#__RefHeading___Toc408535890)

8.2.7 Tumor progression [57](#__RefHeading___Toc408535891)

8.2.8 Unexpected adverse event [58](#__RefHeading___Toc408535892)

8.2.9 Reporting of adverse events [58](#__RefHeading___Toc408535893)

8.2.10 Treatment and follow up of adverse events [59](#__RefHeading___Toc408535894)

8.2.11 Laboratory test abnormalities 59

8.3 Pregnancy 59

9. Independent review committee........................................................................................60

10. Appendix [61](#__RefHeading___Toc408535897)

10.1 General condition (ECOG Performance status) [62](#__RefHeading___Toc408535898)

10.2 Lung cancer international TNM staging classification [62](#__RefHeading___Toc408535899)

10.3 RECIST1.1 tumor response evaluation criteria [65](#__RefHeading___Toc408535900)

10.4 NCI Common Terminology Criteria, Version 4.0 [74](#__RefHeading___Toc408535901)

10.5 Effective treatment of EGFRTKI-related skin adverse events [74](#__RefHeading___Toc408535902)

10.6 Sample analysis [75](#__RefHeading___Toc408535903)

10.7 The New York Heart Association (NYHA) Functional Classification [75](#__RefHeading___Toc408535904)

10.8 Calculation of creatinine clearance [75](#__RefHeading___Toc408535905)

11 References …………………………………………………………………….76

# Abbreviation

AE adverse events

ALT alanine aminotransferase (serum alanine aminotransferase)

AST aspartate aminotransferase (serum aspartate aminotransferase)

CRF case report form

CR complete response

CT computed tomography

CTC-AE common terminology criteria for adverse events

ECG electrocardiogram

EGFR epidermal growth factor receptor

FACT-L functional assessment of cancer therapy- Lung

FISH fluorescence in situ hybridization

HIV human immunodeficiency syndrome

IHC immunohistochemistry

ILD interstitial lung disease

INR international Normalized Ratio

IRC independent review committee

ITT intention to treat analysis

MRI magnetic resonance imaging

NSCLC non-small cell lung cancer

ORR objective response rate

OS overall survival

pCR pathological complete response

PD progressive disease

PFS progression-free survival

PR partial response

PS performance status

QoL quality of life

RECIST response evaluation criteria in solid tumor

SAE serious adverse events

SD stable disease

TKI tyrosine kinase inhibitor

ULN normal upper limit

# Signature

**This clinical trial program——Erlotinib versus gemcitabine plus cisplatin as neoadjuvant treatment for stage IIIA–N2 non-small-cell lung cancer (NSCLC) with EGFR mutation in exon 19 or 21: a multicenter, randomized controlled trial (Protocol ID number：ML25304, C-TONG 1103), is approved to be carried out by the responsible personnel of the following organiztions concurrently.**

Professor Yi-Long Wu

| Principal investigator  Cancer center, Guangdong General Hospital | Signature |  | Date |
| --- | --- | --- | --- |

| Guangdong Association of Clinical Trials | Signature |  | Date |
| --- | --- | --- | --- |

1. **Study background and principle**

## Non-small cell lung cancer

Lung cancer is the firstleading cause of cancer death globally. Non-small cell lung cancer (NSCLC) accounts for 75-80% of all lung malignancies. More than 60% of those patients lost the oppprtunity for sugery or radical radiation therapy at diagnosis^[1]^. Due to the early spread of lung cancer in the lungs, most early stage patients will eventually metastasize and need systemic chemotherapy. Therefore, systemic anti-tumor treatments are playing an increasingly important role in the comprehensive treatment of lung cancer.

The incidence and mortality of malignancies in many countries around the world have increased over the past 30 years. About 25% of lung cancer patients worldwide are in the Asia Pacific region. It is estimated that 50,000 of every 1 million people in China were diagnosed with lung cancer in 2005, and their mortality rate has increased by 1.46 times in the past seven years ^[2]^. Lung cancer was the highest in cancer incidence and has become a powerful killer in the world and in Asia.

When the primary tumor grows in the lung parenchyma or bronchial wall for a time, the tumor will eventually invade the blood vessels and lymphatics and spread through these pathways to regional lymph nodes or to distant metastases. Although lung cancer can metastasis to any organ, the most common organs of metastasis are brain, liver, adrenal gland, bone, and the like.

The only possible effective curative strategy for NSCLC patients is surgical resection, but surgery is only available for a small number of patients with early stage disease. Since most patients have been diagnosed at local advanced or advanced stage, or with poor lung function, less than 30% of patients can receive operation. For many patients with unresectable diseases, radiotherapy can be used to achieve local control, but radical treatment is almost impossible. For locally advanced patients with unresectable tumors, survival can be extended by radiotherapy combined with chemotherapy. For advanced patients who cannot be removed, the main treatment goal is palliative treatment. Patients with unresectable metastatic NSCLC have a poor prognosis with a one-year survival rate of approximately 10% ^[3]^.

## Current status of incidience and treatment of stage ⅢA N2 non-small cell lung cancer

Only about 1/3-1/4 of patients with non-small cell lung cancer can be surgically resected at the time of initial diagnosis. The operable cases include stage I, stage II, and partially resectable stage IIIA patients. For early and partial locally advanced NSCLC, complete surgical resection is the only hope for long-term survival. Patients with stage ⅢA N2accounts for about 15％^[4]^，It contains a broad heterogeneous combination, which is the most challenging and controversial subgroup. Some patients can be cured, while some with poor results is close to patients with stage IIIB and IV. The overall 5-year survival rate is 15-23% ^[5]^.

In the 7^th^ edition of lung cancer TNM staging, in addition to T4N1/T4N0, stage IIIA is characterized by ipsilateral mediastinal lymph node N2 metastasis. The American College of Chest Physicians (ACCP) advocates that N2 can be subdivided into 4 subsets ^[5]^: incidental nodal metastases found on final pathology examination of the resection specimen (IIIA1), nodal (single station) metastases recognized intraoperatively (IIIA2), nodal metastases (single or multiple station) recognized by prethoracotomy staging (mediastinoscopy, other nodal biopsy, or PET scan)(IIIA3) and bulky or fixed multistation N2 disease (IIIA4). This subdivision should be established on preoperative new staging means, such as mediastinum/EBUS or PET/CT. The treatment modes of various subsets are different. Since the 1980s, Pearson and Funatsu have been advocating the effect of mediastinal examination in preoperative diagnosis and induction therapy. They suggested that surgery for patients with N2 metastases should be cautious. From 1992 to 2002, Roth, Rosell and so on underwent 8 phase III clinical trials with small samples, suggesting that preoperative induction chemotherapy for N2 lung cancer patients can increase the surgical resection rate and long-term survival. Theoretically, induction chemotherapy can reduce tumors, improve surgical resection rate, reduce intraoperative dissemination, and inhibit micrometastasis; at the same time, it also has the risk of losing surgery and increasing surgical complications. At present, CT is not considered as a reliable strategy for predicting the nature of mediastinal lymph nodes. It is recommended to perform more objective staging methods such as PET/CT, mediastinoscopy or EBUS for patients with NSCLC before surgery. In general, IIIA1 and IIIA2 are preoperatively diagnosed as cN0 and directly undergo surgery. The current evidence advocates patients with IIIA4 receiving combination of chemotherapy and radiotherapy. Patient with stage IIIA3 N2 was a subgroup of potentially resectable. Mediastinoscopy / EBUS examination can avoid unnecessary open thoracotomy. If patient was diagnosed with N2, they can seek surgery after induction treatment, which will maximize the benefit of patients.

IIIA-N2 non-small cell lung cancer requires multidisciplinary comprehensive treatment cooperation has become a consensus, but the lack of high evidence of fixed treatment mode, which is due to the small number of truly large randomized controlled studies, and relevant research lack of accurate and effective preoperatively staging. The clinical guidelines of lung cancer collaboration organizations all recommended actively carry out registered clinical research in this subgroup. In recent years, the treatment of full-dose concurrent chemoradiotherapy or preoperative induction/radiotherapy in patients with stage IIIA has been basically established based on limited high-quality RCT, but it is also the focus of controversy in different disciplines. According to the new NCCN, for N2 patients confirmed by mediastinoscopy, surgery or radiotherapy may be considered if there is no progress after induction chemotherapy, and adjuvant chemotherapy may be used after surgery. In 2007, Van Meerbeeck et al published a RCT in JNCI, in patients with phase IIIA N2 after induction chemotherapy, surgery or radiotherapy showed no significant difference on PFS and OS ^[6]^. However, for patients with stage IIIA N2 NSCLC who have no progression after induction of radiotherapy, should they choose surgery or definitive radiotherapy？Recently, Albain et al. published a multi-center Phase III RCT-- INT0139 for this issue on lancet ^[7]^. The study enrolled patients with a biopsy confirmed IIIA-N2 stage NSCLC with ipsilateral mediastinal lymph node involvement, in which preoperatively technically resectable clinical N2 patients represented a well-healed subgroup. This study also included some patients with bulky/multi-station N2. This subgroup and IIIB patients were previously selected for clinical studies involving total chemoradiotherapy. This study assumed that the surgery could increase the primary endpoint (2 - year survival rate) from 25% to 35%, while the trial did not eventually reach this aim, which is a relatively high expect in oncology. Although there was no significant difference in 5-year survival between two groups, the absolute value of surgical group increased by 7%. The significance of the study was its secondary endpoint: progression-free survival (surgery vs radiotherapy; 12.8 months vs. 10.5 months; HR 0.77, P=0.017), risk analysis of first progression, and exploratory subgroup analysis on lobectomy or pneumonectomy in surgical group. Distant metastasis remained the most common mode of progression, and result suggested that in selective patients with IIIA lesion who received induction radiotherapy, surgery reduced 12% risk of local recurrence compared with radiation therapy (61 Gy). The incidence of neutropenia and esophagitis in the radiotherapy group was higher than that in the surgical group, but the increase in the local control rate of the surgical group also inevitably paid the cost of treatment-related death (operation vs radiotherapy; 8% vs 2%). Based on this study, will stage IIIA N2 NSCLC undergo surgery after induction of radiotherapy? Eberhardt believes that it can be tried, but should consciously choose patients, such as who need lobectomy instead of pneumonectomy; who are expected to achieve complete resection after induction therapy and have a lower incidence of postoperative complications. For specific patients, it is necessary to weigh the degree of mediastinal invasion, the specific location of the primary cancer, and the number of annual surgery cases and experience of the research center. For stage IIIA N2 NSCLC, clinical studies should be initiated to identify subgroups of patients with potential opportunities for curative treatment. The idea of preoperative molecular targeted induction therapy came into being, according to the experience of induction chemotherapy, and the epidermal growth factor tyrosine kinase inhibitor (EGFR-TKI) in some patients with advanced lung cancer, especially in the first-line treatment of patients with EGFR mutation.

## Background information of erlotinib

Erlotinib is an orally effective epidermal growth factor receptor (EGFR) tyrosine kinase inhibitor (TKI) that targets intracellular region of EGFR tyrosine kinase. As a signal transduction inhibitor, erlotinib can inhibit EGFR downstream signaling pathway including PI3K/AKT, MAPK, STAT3, etc., thereby inhibiting tumor cell proliferation and promoting tumor cell apoptosis. Preclinical studies have shown that erlotinib has a high anti-cancer activity, which can inhibit the growth of a variety of tumors including NSCLC xenograft models.

Erlotinib is an EGFR TKI that is effective in vivo, in vitro and in vitro. The IC50 for inhibiting EGFR phosphorylation in vitro is 2 nM, and the IC 50 for inhibiting EGFR phosphorylation at the cellular level is 16-20 nM. It has obvious anti-tumor, and shows synergistic effect with chemotherapy and radiation therapy.

Up to now, there are currently four phase I clinical trials which evaluate the safety of daily oral and weekly oral erlotinib in patients with advanced cancer. Those study found dose-limiting toxicity when using erlotinib 200 mg/day, mainly included diarrhea and rash; the recommended dose for phase II clinical trial is 150 mg/day, which is the maximum tolerated dose ^[8]^. Herbst et al reported that erlotinib had a half-life of 24.4 hours at 150 mg/day.

## Second or third line erlotinib in advanced NSCLC

Perez-Soler et al explored the efficacy of erlotinib in pre-treated advanced NSCLC, which enrolled patients with EGFR-positive expression. Patients received oral erlotinib 150mg/day to disease progression or 52 weeks ^[9]^. Among 57 patients, 60% were women, with a median age of 62 years and a PS 0-1 of 77%. EGFR was strongly expressed in 32 cases, moderate expression in 19 cases, and mild expression in 6 cases. The response rate of this group was 12.3%, including 2 cases of CR and 5 cases of PR; the disease stability rate was 36.8%. Median duration of remission was 19.6 weeks; median survival was 8.4 months; 1-year survival rate was 40%; and median PFS was 9 weeks. The response rate of patients who received ≥ 2 chemotherapy regimens was 12.8%, suggesting that the number of chemotherapy regimens did not affect the efficacy of erlotinib. Of the 15 patients who had treated with taxotere, 4 achieved CR or PR and 5 had SD; although those patients had a high response rate, overall survival (8.6 months) and 1-year survival (47%) were similar with overall patients. The incidence of fatigue, dyspnea, and cough decreased from 67%, 61%, and 60% before treatment to 49%, 37%, and 39%. The main adverse reactions were rash and diarrhea, similar to the results in phase I clinical trials. The incidence of rash was 67%, diarrhea was 56%, skin was dry at 35%, itching was 35%, and nausea was 25%. It also found that the expression level of EGFR was not associated with the short-term efficacy of erlotinib. The median time to rash was 10 days and the median time to diarrhea was 14 days.

Miller et al ^[10]^ reported the preliminary results of erlotinib in 50 patients with bronchoalveolar cell carcinoma (BAC). The median age of those patients was 66 years and 68% of women, 75% of patients had a history of smoking, and 76% had no chemotherapy. The response rate was 26%, and erlotinib was well tolerated. The main adverse events included rash (12%) and diarrhea, and no grade 4 adverse reactions were reported.

Multi-centre phase III trial BR. 21 compared the efficacy and safety of seconde or third line of erlotinib and placebo in advanced NSCLC ^[11]^. The study enrolled patients with PS 0-3, who had failed first- or second-line chemotherapy, and a total of 731 patients were included. It first found that erlotinib significantly prolonged overall survival of advanced NSCLC compared with placebo, with a median survival improvement of 42.5% and a 1-year survival rate of 45%, both exceeding the initial assumption of 33%; it also significantly improves the quality of life. Survival benefit was found in multiple subgroups, and efficacy was not affected by smoking status, histological type, gender, previous chemotherapy regimen, and physical status (PS). Adverse reactions were similar to those in Phase II and Phase I clinical trials, suggesting that second and third-line erlotinib in advanced NSCLC had better safety and tolerability. Further large-scale TRUST tests confirmed the results of BR.21 later.

A subgroup analysis of the BR.21 trial showed that erlotinib was effective in Asian patients, with a median survival of 13.8 months, an increase of 5.2 months compared with 8.6 months in the placebo group, and decrease 40% risk of death. Interim analysis of TRUST trials evaluated more than 1,200 from five regions of East and Southeast Asia, showed that erlotinib has better efficacy in advanced NSCLC in second-line or third-line treatment. The objective response rate is about 24%, and the disease stability rate is 54%, disease control rate is up to 78%; median progression-free survival was about 5.52 months. The main adverse events were rash and diarrhea, mainly mild to moderate. The incidence of serious adverse events related to erlotinib was only about 3 to 5%; rate of rare adverse events of erlotinib was 19%, the vast majority of which were grade 1 to 2.

## Erlotinib in EGFR mutant NSCLC

EGFR is a tyrosine kinase receptor, located in the p13-q22 region of chromosome 7 and has a full length of 200 kb. It consists of 28 exons and encodes 1186 amino acids ^[12]^. Its glycoprotein has a molecular weight of approximately 170 kDa ^[13]^, widely distributed in all tissue cells except mature skeletal muscle cells, body wall endoderm and hematopoietic tissue. EGFR family has four structurally similar receptor molecules: ErbBl (EGFR), ErbB2 (HER2), ErbB3 (HER3), ErbB4 (HER4), which belong to the receptor tyrosine kinase (RTKS). They all contain an extracellular ligand binding domain, a transmembrane domain and a cytoplasmic domain with tyrosine kinase activity. Its intracellular region is highly homologous to the erbB oncogene product ^[14]^.

Abnormal EGFR activation mechanisms include gene amplification, overexpression of receptor and ligands, activating mutations, and lack of a negative regulatory pathway. Hence, EGFR induces cancer through at least three mechanisms: overexpression of EGFR, EGFR gene amplification or mutation ^[15]^. Among these three mechanisms, EGFR activating mutation is the most important factor leading to abnormal biological behavior of tumor cells.

Studies found that approximately 10% of NSCLC patients treated with tyrosine kinase inhibitor gefitinib or erlotinib showed rapid and satisfactory clinical results. Further studies found that most of these patients had EGFR mutation. Mutations known to be associated with EGFR-TKI (endothelial growth factor receptor tyrosine kinase inhibitor) included the following: G719X (exon18), E746-A450 deletion (exon19), L858R (exon21), L861Q (exon 21), T790M (exon20) and D770-N771 (exon 20). Of which, E746-A450 deletion and L858R are highly related to the efficacy of TKI. Mitsudomi T. Yatabe Y analyzed 568 non-small cell lung cancer patients: about 90% of EGFR mutations were in exon 19 or 21. EGFR-TKI in patients with exon 19 deletion and exon 21 point mutations achieved ORR of more than 70% ^[1^**^6^**]. Moreover, the rate of EGFR mutation in Asian population is significantly higher than that in the Caucasian population.

SLOG study conducted by Rosell is a phase II, prospective, Spanish multicenter clinical trial ^[17]^. This study prospectively evaluated the feasibility of large-scale EGFR mutation testing and the correlation between mutation and long-term or short-term efficacy of erlotinib. A total of 2105 patients were enrolled in the study, and it used microdissection and standard sequencing. It found that 350 patients with EGFR mutations, of which 217 EGFR mutated patients received erlotinib, with a median PFS of 14 months, a median survival of 27 months, and a complete response rate (CR) of 12.2%, partial response rate (PR) was 58.4%, and objective response rate (ORR) (complete remission plus partial remission) was 70.6%, which was significantly higher than that of traditional chemotherapy.

OPTIMAL is a phase III, prospective, multi-center trial, which compared the efficacy of first-line erlotinib and carboplatin/gemcitabine in Chinese NSCLC patients with EGFR mutation. Erlotinib significantly prolonged the primary endpoint of PFS comparing with chemotherapy (13.1m vs 4.6m, HR=0.16, p <0.0001), in addition, the objective response rate and disease control rate of erlotinib were aslo significantly higher than that in chemotherapy (83% vs36%，p=0.0000；96% vs 82%，p= 0.002) ^[23]^.

### Rationale of dose selection of erlotinib

Erlotinib 150 mg/day was based on pharmacokinetic parameters, safety and tolerability observed in phase I, II, III studies in advanced refractory cancer patients. Among cancer patients with a therapeutic dose of 150 mg/day, blood level is consistently higher than the average blood concentration of 500 ng/mL, which can achieve clinical efficacy.

## Rationale of study

This study hypothesized that in IIIA-N2 EGFR activating mutant non-small cell lung cancer (NSCLC), the objective response rate and pathological N-stage down-regulation rate of EGFR-TKI neoadjuvant therapy is superior to traditional platinum-containing chemotherapy regimen. This assumption is based on the following two principles.

### The efficacy of EGFR-TKI and traditional platinum-containing chemotherapy in lung cancer patients with EGFR activating mutation

IPASS study compared first line gefitinib and carboplatin/paclitaxel in Asian patients with lung adenocarcinoma. Among 261 patients with EGFR mutation, the PFS of gefitinib was significantly longer than chemotherapy (HR 0.48, p <0.001) ^[18]^.

The Spanish lung cancer group ^[17]^ SLCG enrolled more than 2000 advanced NSCLC patients, and 217 patients with EGFR mutations had evaluable efficacy, of which first-line treatment accounted for 52%. The results showed complete remission rate of 12.2%, ORR of 70.5%, median disease progression time (TTP) of 14 months, OS of 27 months. Compared with the results of conventional first-line chemotherapy reported in literature, erlotinib in EGFR mutant advanced NSCLC could improve the efficacy by 2-3 times when compared with chemotherapy, which confirmed the results of biomarkers analysis in IPASS study.

The OPTIMAL study in section 1.3.2 showed that the PFS in erlotinib was 3 times longer than that in chemotherapy group, which also indicating the efficacy of EGFR-TKI was superior to chemotherapy in EGFR mutant NSCLC.

### Potential benefit of pre-operative introduction target therapy

Compared with neoadjuvant chemotherapy, neoadjuvant target therapy can shorten the time of preoperative therapy, decrease the toxicity of preoperative treatment and surgical complications, increase the tolerance of surgery, and cure tumor by the concept that sensitive clones killed by EGFR-TKI rather than by surgical removal. This study enrolled patients with IIIA-N2/II phase-N1 with intrathoracic lymph node metastasis and EGFR mutations. It is expected that the response rate of EGFR-TKI induction therapy will significantly improved the efficacy compared with traditional chemotherapy. Several studies suggested the feasibility of this treatment strategy, such as several case reports ^[21]^ of surgery after targeted therapy in patients with EGFR mutation N2 published in lung cancer ^[19]^ and JCO et al ^[20]^ since 2007, and phase II study of Tarceva in III N2 NSCLC (CSLC 0702, NCT00600587)^[22]^. However, no high level of prospective randomized controlled trial has been published.

## Questions to be addressed

Main issues that still need to be addressed today: How effective is targeted therapy? What is the time schedule of TKI targeted induction therapy? How to choose the target population? Does EGFR-TKI affect postoperative wound healing and recovery? Does targeted induction therapy improve postoperative progression-free survival and overall survival? How to use molecular biology for guiding further treatment after post-operative tumor recurrence? In addition, the mechanism of EGFR-TKI resistance is hot area of research. It is expected to obtain tissue from the same individual at multiple time-points, and multiple tumor samples from different sites (primary lesions, mediastinal lymph nodes) at each time-point in this study. It would help us understand the dynamic changes of some related gene mutations. Furthermore, the dynamic changes of the EGFR and KRAS genes in tumor tissue, paracancerous tissue and blood taken at three time points: mediastinal examination, radical surgery and tumor progression will elucidate the heterogeneity of EGFR mutations.

1. **Study objectives**

The primary objective of this study was to compare the efficacy and safety of erlotinib versus gemcitabine/cisplatin neoadjuvant therapy in surgically resectable IIIA-N2 with epidermal growth factor receptor (EGFR) exon 19 or 21 sensitive mutant non-small cell lung cancer (NSCLC).

## Primary objective

To compare the objective response rate (ORR) of erlotinib versus gemcitabine/cisplatin neoadjuvant therapy in surgically resectable IIIA-N2 with epidermal growth factor receptor (EGFR) exon 19 or 21 sensitive mutant non-small cell lung cancer (NSCLC).

## Secondary objectives

• Compare lymph node downgrade rate of 2 treatment groups after neoadjuvant therapy;

• Compare complete resection rate of 2 treatment groups after neoadjuvant therapy;

• Compare pathologic complete remission (pCR) of 2 treatment groups after neoadjuvant therapy;

• Compare progressive free survival (PFS) of 2 treatment groups after neoadjuvant therapy;

• Compare 3-year overall survival rate (3-year OS rate) and 5-year overall survival rate (5-year OS rate) of 2 treatment groups after neoadjuvant therapy;

• Compare safety of 2 treatment groups using NCI CTC-AE (Version 4.0);

• Compare quality of life (QoL) of 2 treatment groups.

## Explortary analysis

• Post-operative 24 week, 48 week and 3 year disease free survival (DFS) rate;

• Explortary biomarker analysis. Potentinal biomarkers included T790M, cMET, KRAS, ALK-EML4, BRAF, PTEN.

1. **Study design**

## Study design and planning

This is a prospective, open-label, randomized, controlled, multi-center trial, aiming to compare the efficacy and safety of erlotinib versus gemcitabine/cisplatin neoadjuvant therapy in surgically resectable IIIA-N2 with epidermal growth factor receptor (EGFR) exon 19 or 21 sensitive mutant non-small cell lung cancer (NSCLC). It intends to enroll 72 patients, randomized to receive 6 weeks of erlotinib neoadjuvant therapy or 2 cycles of gemicitabine/cisplation. After neoadjuvant therapy, surgical resectabe patients by investigators will receive surgery. At least 6 stations of lymph nodes should be removed during surgery: 3 stations of N2 lymph nodes (including subcarinal lymph nodes) and 3 stations of N1 lymph nodes.

It is recommended that all surgical patients receive postoperative adjuvant therapy within 8 weeks after surgery (erlotinib group receive erlotinib for 1 year, chemotherapy group receive 2 cycles of gemicitabine and cisplatin). Patients who achieve benefit from neoadjuvant therapy (CR, OR, SD without enlarge and enlarged SD with newly emerged tumor necrosis) will receive the adjuvant therapy showed above. If patients achieve enlarged SD without newly emerged tumor necrosis and undergo surgery, or achieve PD after neoadjuvant therapy and undergo surgery (i.e., satellite lesions appear in ipsilateral thoracic cavity or the same lung lobe), the investigator will determine the adjuvant treatment strategy.

Investigators at each center can decide whether to give postoperative adjuvant therapy to patients with stage IA or pathologic complete remission (pCR) based on specific conditions, but it is recommended to provide postoperative adjuvant therapy to all patients. If patient's surgical margin has residual tumor (R2) or microscopic residual tumor (R1), it is recommended that patients undergo postoperative radiation therapy. In addition, for patients diagnosed with N2 by mediastinoscopy before treatment, mediastinoscopy may cause adhesion and fibrosis of lymph node drainage; hence the difficulty of lymph node dissection will increase. Surgeons should decide whether the patient receives postoperative radiotherapy according to the specific conditions of lymph node dissection. Patients in erlotinib group can receive erlotinib during radiotherapy concurrently; it is recommended that post-operative radiotherapy and adjuvant chemotherapy should undergo sequentially.

## 3.2 Randomization

This is a randomized controlled study, and patients will be randomized into 2 groups as following:

Erlotinib group: receiving erlotinib as pre-opearative neoadjuvant therapy and post--opearative adjuvant therapy.

Chemotherapy group: receiving gemicitabine and cisplatin as pre-opearative neoadjuvant therapy and post--opearative adjuvant therapy.

## 3.3 Patients selection

Eligible patients included previously untreated resectable stage IIIA-N2 NSCLC, and tumor tissue testing showed EGFR exon 19 or 21 mutation. The specific meaning of surgical resectable was technically resectable by investigators.

N2 disease confirmed by mediastinoscopy, endobronchial ultrasound (EBUS), PET/CT or thoracoscope.

The time of stage IIIA-N2 diagnosis was within 28 days prior to randomization.

## 3.3.1 Inclusion Criteria

**Inclusion criteria related to disease:**

- Histologically or cytologically confirmed NSCLC;
- Previously untreated with operation, chemotherapy, biotherapy and radiotherapy; the specific meaning of surgical resectable was technically resectable by investigators.
- Gene testing of tumor tissue from primary tumor or metastatic lymph nodes confirmed EGFR exon 19 or 21 mutation. N2 disease confirmed by mediastinoscopy, endobronchial ultrasound (EBUS), PET/CT or thoracoscope.
- Sufficient tumor tissue (not cytology specimens) available for molecular marker analyses;
- At least one measurable lesion with a largest diameter of ≥10 mm on spiral CT according to Response Evaluation Criteria in Solid Tumors version 1.1 (RECIST 1.1).

**Hematology, biochemistry and organ function:**

- Pulmonary ventilation function, FEV1≥1.5L or anticipated FEV1≥800ml after lobectomy or pneumonectomy;

(The following laboratory tests performed with 7 days prior to the first dose confirmed that patients’ bone marrow, liver and kidney function met the study requirement:);

- Hemoglobin ≥ 9.0 g/dL (can be maintained or exceed by blood transufusion);
- Absolute neutrophil count ≥1.5×10^9^/L；
- Blood platelet count ≥100×109/L；
- Total bilirubin ≤1.5 times the upper limit of normal;
- AST and ALT ≤2.5 times the upper limit of normal;
- Creatinine ≤1.25 times the upper limit of normal; and creatinine clearance rate ≥60mLl/min;
- International normalized ration (INR) of prothrombin time in patients without anticoagulant therapy ≤1.5, partial thromboplastin time (APTT) ≤1.5 times the upper limit of normal. Patients treated with full dose or parenteral anti-coagulation treatment were eligible if the dose of anticoagulant was stable for at least 2 weeks before enrolling the clinical trial and the result of blood conagulation test belonged to the range defined by the local laboratory；
- Child-bearing women (15-49 years) should have urine pregnancy test within 7 days before treatment, and the result should be negative.

**General inclusion criteria:**

- Provision of signed informed consent by patients or their legal representative;
- Patients received oral drugs according to study protocol and follow-up process;
- Patients aged ≥18 years old;
- Patients can tolerate neoadjuvant therapy (including neoadjuvant chemotherapy) and operation, ECOG performance status 0-1, life expectarncy >12 weeks;
- Men and women of childbearing age agreed to adopt a reliable method of contraception before enrolling the trial, during the research and within 30 days after stopping the drug.

## 3.3.2 Exclusion criteria

- Any previous systemic anti-cancer cancer therapy for NSCLC, including cytotoxic therapy, target therapy (including tyrosine kinase inhibitor or monoclonal antibody) and experimental study;
- Pevious local radiotherapy for NSCLC;
- Patients with preious malignancies other than NSCLC within 5 years, except for cured in situ carcinoma of the uterine cervix, cured basal cell carcinoma and bladder epithelial tumor [including Ta and Tis];
- Any unstable systemic disease, including active infection, uncontrolled hypertension, unstable angina, angina that began to attack in the last 3 months, congestive heart failure (≥ New York Heart Association [NYHA] class II), myocardium infarction (6 months befor enrollment), severe arrhythmia requiring medication, liver, kidney or metabolic disease；
- Previously or currently with interstitial lung disease;
- Incomplete or uncontrolled eye inflammation or eye infection, or any condition that may cause the above mentioned eye diseases;
- Confirmed human immunodeficiency virus (HIV) infection;
- Allergic to erlotinib or gemcitabine or cisplation;
- Patients who underwent major operation or severe trauma withion 2 months prior to the first dose;
- Patients with small cell lung cancer;
- Pregnant or lactating women;
- Investigartors evulate that patients were not suitable for enrollment, such as neurological or metabolic disorders, physical examination or laboratory suspecting that patients have a possible disease or have contraindications to study drug use, or has a high risk of treatment-related complications.

## 3.4 Study period

Each enrolled patient had a neoadjuvant treatment period for approximately 2 months, operation and post-operative recovery period for about 1 months, follow-up for 2 years after operation and then survival follow-up for 3 years. In total, the longest study time for every enrolled patient was about 64 months.

Calculated according to approximately 5 patients per month, this study intended to enroll 72 patients, and it would takes 18 months for enrollment. Patients will be follow-up for 2 years after operation and then had survival follow-up for 3 years. The total study period takes approximately 82 months.

## 3.5 Patients withdraw from study

Withdraw patients mean that patiens discontinue study drugs for various reasons. Patients who have the following conditions will withdraw from the study:

- - - Patient himself or his legal representative requests to withdraw;
    - According to investigator’s suggestion, patients who continue to participate in the study will be harmful to their health;

Patients can withdraw at any time during the trial. Patients have right to withdraw from the study at any time for any reason. If patients decided to withdraw from study treatment, investigators should try best effort to collect and report results. Investigators should contact patients or their relatives by phone or in persion to clarify the reasons for patients’ withdraw. If patients withdraw from study due to adverse events, investigators should record the primary related adverse eventson medical report form. If patients decided to stop treatment early, or treatment stopped early based on investigators’ suggestion, investigators should consult with patients to determine if they can continue to contact him/her to obtain more relevant information for completely survival follow-up. The results of discussion should be recorded in medical history and CRF.

The investigor believe that patients must withdraw from study in the following circumstances:

- - - Receing other anti-cancer treatment during study period;
    - Severe allergic reations to study drug, such as exfolative rash or grade 3-4 hypersensitivity reaction;
    - Any other serious adverse events deemed by investigators that needed durg discontinuation;
    - Patients with severely poor adherence；
    - Patient’s urine β-HCG test indicated pregnancy. Investigators should report the pregnancy status of this patient in form of clinical trial pregnancy report form;
    - Patient has other disease concomitantly, and investigator believe this disease would significantly affect the assessment of patient’s clinical condition and need to stop the study drug;
    - Patients has other malignancies that requires treatment;
    - Patients who lost follow-up;
    - Patient uses illicit drugs or other substances that may cause toxic reactions or bias the results of study, as judged by the investigator;
    - Chemotherapy delayed over 21 days; or erlotinib discontinued over 2 weeks due to adverse events;
    - Patient died.

All patients who withdraw from the study should record the reason for withdrawal in case report form and this patient’s medical record.

Enrolled patients who cannot receive study drug for various reasons, will receive treatment recommended by the investigator based on the latest clinical guidelines.

Patients who withdraw due to adverse events or laboratory abnormality should be followed up until adverse events have stabilized, and the subsequent outcome of events should be recorded. If patients died within study period or 28 days after study completion, the investigator must record the cause of death on severe adverse events (SAE) report form within 24 hours.

## 3.6 Treatment plan

## 3.6.1 Treatment arrangement

**Neoadjuvant therapy**

**Erlotinib group**: erlotinib 150 mg/day administered orally for 6 weeks (42 days), treated until disease progression or unacceptable toxicity.

**Chemotherapy group:** gemcitabine 1250mg/m^2^ administered intravenously on day 1 and day 8.

Cisplatin 75 mg/m^2^ administered intravenously on day 1.

Every 3 weeks for 2 cycles, treated until disease progression or unacceptable toxicity.

**Surgery**

**Eroltinib group:** Tumor efficacy was evaluated by CT or PET/CT scan with 1 week after 6 weeks of neoadjuvant therapy (the 7^th^ week, day 43 to 49). After neoadjuvant therapy, surgically resectable patients by inverstigators undergo surgery.

**Chemotherapy group:** Tumor efficacy was evaluated by CT or PET/CT scan with 1 week after 2 cycles of neoadjuvant therapy (the 7^th^ week, day 43 to 49). After neoadjuvant therapy, surgically resectable patients by inverstigators undergo surgery.

Surgically resectable patients underwent surgery at the 8^th^ (day 50 to 56) or 9^th^ (day 57 to 63) week. At least 6 stations of lymph nodes should be removed during surgery: 3 stations of N2 lymph nodes (including subcarinal lymph nodes) and 3 stations of N1 lymph nodes.

**Post-operation**

It is recommended that all surgical patients receive postoperative adjuvant therapy within 8 weeks after surgery (erlotinib group receive erlotinib for 1 year, chemotherapy group receive 2 cycles of gemicitabine and cisplatin). Patients who achieve benefit from neoadjuvant therapy (CR, OR, SD without enlarge and enlarged SD with newly emerged tumor necrosis) will receive the adjuvant therapy showed above. If patients achieve enlarged SD without newly emerged tumor necrosis and undergo surgery, or achieve PD after neoadjuvant therapy and undergo surgery (i.e., satellite lesions appear in ipsilateral thoracic cavity or the same lung lobe), the investigator will determine the adjuvant treatment strategy.

Investigators at each center can decide whether to give postoperative adjuvant therapy to patients with stage IA or pathologic complete remission (pCR) based on specific conditions, but it is recommended to provide postoperative adjuvant therapy to all patients. If patient's surgical margin has residual tumor (R2) or microscopic residual tumor (R1), it is recommended that patients undergo postoperative radiation therapy. In addition, for patients diagnosed with N2 by mediastinoscopy before treatment, mediastinoscopy may cause adhesion and fibrosis of lymph node drainage; hence the difficulty of lymph node dissection will increase. Surgeons should decide whether the patient receives postoperative radiotherapy according to the specific conditions of lymph node dissection. Patients in erlotinib group can receive erlotinib during radiotherapy concurrently; it is recommended that post-operative radiotherapy and adjuvant chemotherapy should undergo sequentially.

**Erlotinib group:** If investigators considered post-operative adjuvant thearapy, then patients would receive erlotinib for 12 months after surgery or until disease progression or unacceptable toxicity. Erlotinib 150 mg/day administered orally.

**Chemotherapy group:** If investigators considered post-operative adjuvant thearapy, then patients would receive chemotherapy for 2 cycles (every 3 weeks for 1 cycle) after surgery or until disease progression or unacceptable toxicity.。

Gemcitabine 1250mg/m^2^ administered intravenously on day 1 and day 8.

Cisplatin 75 mg/m^2^ administered intravenously on day 1.

All patients who underwent surgery were followed up for 2 years after surgery, and the follow-up schedule within 2 years were as follows:

- If patients received adjuvant treatment after surgery, a chest-enhanced CT examination should be performed within 28 days before the start of the adjuvant treatment. Postoperative follow-up visits were performed every 3 months from the first day of adjuvant therapy until disease recurrence. If patients did not receive adjuvant treatment after surgery, a chest-enhanced CT examination should be performed within 8 weeks. After that, patients would be follow-up every 3 months until disease recurrence. The follow-up included patients’ general condition, symptoms and signs, blood routine, biochemical routine test, thoracic-enhanced CT and abdominal B-ultrasound (including liver, gallbladder, spleen, pancreas, kidneys, and bilateral adrenal glands). Collecting blood samples at each follow-up for explortary biomarker analysis; if patients recurred after 2 years post-operation, collecting blood sample at recurrence.
- Cranial-enhanced MRI scan every 6 months.
- Bone scan every 12 months.

If patients recurred, then patients would be followed up every 3 months until 5 years after surgery or death.

If patients progressed after neo-adjuvant therapy and could not undergo surgery, then patients would be followed up every 3 months until 5 years after randomization or death.

## 3.6.2 Auxiliary medicaiton

Gemcitabine combined with cisplatin may cause sever bone marrow suppression. Granulocyte colony-stimulation factor (GCSF) or granulocyte/macrocellular colony-stimulating factor (GMCSF) should not be used preventionly in the first cycle, but it can be used in the following cycles. It is recommended to use granulocyte colony-stimulation factor according to the guidelines of American Society of Clincal Oncology (ASCO).

## 3.6.3 Delay in drug delivery and dose adjustment of chemotherapy drugs

The dose of gemcitabine and cisplatin on the first day of each cycle should be determined according to body surface area in neoadjuvant and adjuvant period; the dose of gemcitabine on the eighth day of each cycle should be adjusted according tohematological toxicity.

Patients’ toxicity should be carefully monitored. The dose of gemcitabine or cisplatin should be adjusted according to patients’ tolerance. It is recommended to make dose adjustments on the most severe toxicities and laboratory abnormalities that occurred in the past cycle or the first 7 days of this cycle. Toxic reaction should be graded according to NCI CTC-AE 4.0. If patients had several adverse events concurrently, then dose adjustment should be made based on the most severe toxicity. Do not increase the dose of gemcitabine or cisplation during chemotherapy phase.

The maximum time allowed for treatment delay is 3 weeks, and symptoms are required to be reduced to CTC-AE level 1 or below during this period. For patients with grade 2 or more severe CTC-AE at baseline, then symptoms are required to be reduced to baseline level.

- - - Drug can be reduced/suspended due to adverse events at any stage of the study.
    - If patients has discontinued gemcitabine plus cisplatin for more than 3 weeks due to treatment-related adverse events, and cannot control symptoms even after propoer symotomatic treatment, they should withdraw from the study.
    - If patients needed to be admitted to hospital during chemotherapy period due to treatment-related adverse events, all study drugs (gemcitabine and cisplatin) should be discontinued before all drug-related symptoms recoveried.
    - Adverse events should be graded by NCI CTC-AE 4.0 (see section 10.4).
    - If patients needed to reduce more than 3 levels of dose due to toxicity, then they should be withdrawn.

#### 3.6.2.1 Dose adjustment

In the chemotherapy phase, the dose of gemcitabine and cisplatin should be reduced or discontinued when patients had serious toxicity caused by either of the drugs.

In the chemotherapy phase, the dose in next cycle will be adjusted according to toxicity of the previous cycle. Once the dose has reduced, the dose of reduced drug will not allow for increasing to the original dose. Do not increase the dose of gemcitabine or cisplation during chemotherapy phase.

- - - If patients had severe hypersensitivity reactions to gemcitabine or cisplatin during treatment, drugs should be stopped immediately and record this adverse event.
    - If patients had severe hypersensitivity reactions on the first day of cycle 1, then patients should be excluded from the study.
    - If patients had severe hypersensitivity reactions to gemcitabine or cisplatin and finished 2 cycles of treatment, then investigators shoud also record imaging response, SD, PR or CR based on RECIST 1.1.

#### 3.6.2.2 At the beginning of next chemotherapy cycle, laboratory results must meet the following criteria.

- - - Neutrophils ≥1.5×10^9^/L,
    - Platelets ≥100×10^9^/L,
    - Blood creatinie ≤1.25×ULN,
    - Bilirubin ≤ 1.5×ULN,
    - AST and ALT≤2.5×ULN

If patients’ laboratory results did not recover after 21 days of delay, then patients should withdraw from the study.

#### 3.6.2.3 Dose adjustment of gemcitabine on day 8 (Neutral granulocyte, platelet, non-hematologic toxicity, any of which meet the criteria, then patients should adjust the dose)

| Neutral granulocyte  （10^9^/L） | Platelet  （10^9^/L） | Non-hematologic toxicity  （except for nausea, vomiting and hair loss） | Dose of gemcitabine |
| --- | --- | --- | --- |
| ≥1.5 | ≥75 | G1 | 100% |
| 1.0～1.49 | 50～74 | G2 | 75% |
| <1.0 | <50 | G3～4 | 0% |
| Neutropenia fever |  |  | 0% |
| Please note:   - If patients had severe (grade 3 or 4 ) non-hematologic toxicity (except for hair loss, nausea/viomting), gemcitabine on day 8 should be stopped. - If patients had fever (>38.5℃), confired infection or bleeding, gemcitabine on day 8 should be stopped. - If adjusting the dose of gemicitabine on day 8 has no effect on the administration of gemcitabine and cisplatin in the next cycle, then it can be adjusted. - If the indicators specified in Table 4 did not restored with 7 days, then chemotherapy will be stopped for once. | | | |

#### 3.6.2.4 Dose of gemcitabine and cisplatin in next cycle shoule be adjusted based on toxic reactions in the past cycle：

Any of which occurred in the past cycle, dose will be reduced by 25% in next cycle:

- - - Neutrophils ＜0.5×10^9^/L
    - Neutrophils with fever 38.5℃
    - Neutrophils with comfirmed fever
    - Platelet count ＜25×10^9^/L
    - Platelet count ＜50×10^9^/L with bleeding
    - In addition to skin reactions and untreated nausea and vomiting, dose of gemcitabine and cisplatin should be reduced based on the levels in the below table if patients had other non-hematologic toxicities. Nausea and vomiting that cannot be relieved after treatment, dose of gemcitabine and cisplatin should also be reduced according to the methods listed in the below table.

| Table: dose adjustment in the presence of non-hematologic toxicity | |
| --- | --- |
|  | Grade 3 to 4 adverse events |
| Gemcitabine | Stop drug ^a^ until the dose reaction reduced to ≤ grade 2, then reduce drug by 25% |
| Cisplatin | Stop drug ^a^ until the dose reaction reduced to ≤ grade 2, then reduce drug by 25% |
| a If toxic reactions could not recoverd after 21 days of drug discotinuation, then patients should withdraw from study. Toxic reactions graded by NCI CTC-AE 4.0. | |

## 3.6.3 Delay in administration and dose adjustment of erlotinib

The dose of erlotinib was adjusted according to rash and other toxic reactions.

Based on the results from BR.21 and TRUST, singal agent of erlotinib was tolerated well, and about 13% of patients need drug reduction, 3% of patients discontinued the drug for adverse events. The main reasons for discontinuation and dose reduction were rash and diarrhea. In the following situations, dose reduction or discontuation need to consider:

- - - Grade 3-4 rash (refer to NCI CTC-AE 4.0), no relief after medical treatment. After drug discontinuation and rash recoveried to below grade 1, restart treatment can be considered, while the dose should be reduced by 50mg.
    - Grade 3-4 diarrhea, no relief after medical treatment, drug should be discontinued. After completely recovery, restart treatment can be considered, while the dose should be reduced by 50mg.
    - Drug should be discontinued immedicately when suspect of interstitial lung disease (ILD). Once diagnosed, erlotinib should be stopped forever. If ILD was excluded, patients can consider restart treatment.
    - Other grade 3-4 adverse events, such as abnormal liver function, oral ulcer, etc., drug should be discontinued. After adverse events recoveried to below grade 1, dose should be reduced by 50mg for restart.
    - Reduce to a minimum dose of no less than 50 mg, otherwise withdraw from the study.

## 3.6.4 Concomitant treatment and smoking

All concomitant medications and treatment (including start/stop dates and indications) must be recorded in patients’ original data and in the corresponding section of case report form (CRF).

Erlotinib is primarily metabolized by CYP3A4 in cytochrome P450, and only a small part metabolized by CYP1A2. Therefore, all patients who take drugs that are metabolized by CYP3A4 need to closely observe for possible adverse reactions of those drugs.

Smoking can affect the pharmacokinetics of erlotinib. During treatment, patients’ smoking status should be recorded, including the number and duration of smoking per day. All patients are advised to quit during treatment.

## 3.6.5 Concomitant medication

#### 3.6.5.1 Drugs permitted

- - - Bevacizumab and any drug that targets VEGF, VEGFR or EGFR (including registered or study drug) .
    - Except for the designated gemcitabine, cisplatin and erlotinib, any other anticancer drug is not allowed, including study drugs (eg, research antibiotics, antiemetics, etc.) and Chinese herbal which has anti-cancer indications.

#### 3.6.5.2 Drugs prohibited

- - - Non-anti-cancer Chinese herbal, acupuncture, vitamins/microelements are allowed to be use without affecting the study endpoint, and the investigator should control those conditions.
    - Patients can receive palliative care and supportive care for their original disease.

## 3.6.6 Treatment compliance

The dose and date of administration of gemcitabine and cisplatin used in each cycle per patient should be recorded in CRF. The dose and date of administration of erlotinib used in each cycle per patient should be recorded in CRF. The cause of delay dosing, drug reduction or miss should also be recorded in the CRF.

Patients’ adherence to treatment and protocol means voluntary compliance with all aspects of the protocol, including compliance with durg, all blood collection required for safety evaluation, regular follow-up after sugery. Patients who do not take medication on time, do not cooperate with the examination, or do not return on time, may be excluded from the study according to the main investigator's opinion. If patients stopped erlotinib because of adverse events alleviate even giving the optimal symptomatic and supportive treatment, and the time of stopping has lasted for > 2 weeks, the patient withdrew from the study after discussing with and obtain agreement from principal investigator.

## 3.7 Study endpoints

The primary endpoint of this study is objective response rate (ORR).

Secondary endpoint includes: downsating rates of pathological lymph nodes, pCR, PFS, 3 year and 5 year OS rate, quality of life (QoL) and safety biomarkers.

Exploratory analysis: Post-operative 24 week, 48 week and 3 year disease free survival (DFS) rate, exploratory biomarker analysis (potentinal biomarkers includes: T790M，cMET, KRAS, ALK-EML4, BRAF, PTEN).

### 3.7.1 Efficacy endpoint

Objective response rate (ORR) defined as the proportion of patients whose tumor shrink to a certain amount, it includes patients with complete response (CR) and partial Response (PR).

Complete resection rate defined as the proportion of patients who received completely resection (R0 section).

Downsating rates of pathological lymph nodes defined as the proportion of patients with pathological confirmed lymph nodes downstaging from N2 to N1 or N0 after neoadjuvant treatment.

Pathological complete response (pCR) rate defined as the proportion of patients with complete disappearance of tumor cells in all pathological specimens.

Progression free survival (PFS) defined as the time from randomization to the first confirmed disease progression, relapse or death from any cause, whichever occurs first. For patients underwent surgery, if patient had no disease recurrence or death at the end of the study (or data cut-off), or patients who were lost after surgery were truncated on the date of final confirmed recurrence-free. In patients who did not undergo surgery, the tumor assessment after the end of neoadjuvant therapy was considered as an event for patients with disease progression; patient without disease progression was censored at the time of disease progression after neoadjuvant therapy. Patients who had not undergone a tumor assessment after baseline, the time of randomization was considered as cut-off date.

3-year OS rate and 5-year OS rate：OS defined as the time from randomization to death of any cause. The 3-year overall survival rate refers to the probability that patient will live at 3 years after randomization. The 5-year overall survival rate refers to the probability that the patient will live at 5 years after randomization.

**24-week, 48-week, 3-year DFS rate:** DFS defined as the time from surgery to disease progression or death of any cause (which comes first). 24-week， 48-week，3-year DFS rate refers to the probability that patients was recurrence-free at 24-week, 48-week and 3-year.

Investigators will evaluate tumor response and disease progression based on RECIST 1.1.

For patients who survived and did not have disease recurrence at the deadline, the time of their last imaging assessment will be choosed as cut-off date.

## 3.7.2 Patients’ self-assessment

QoL and lung cancer symptoms will be assessed by function assessment of cancer therapy - lung (FACT-L), 4th edition and lung cancer symptom scale (LCSS). Patients should complete FACT-L and LCSS scales independently at the beginning of each visit, before the conversation between patient doctor, which will ensure that any contact between patient and physician does not affect the response to the questionnaire.

FACT-L questionnaire and LCSS scale evaluation in pre-operation: patients in tarceva group complete the evaluation on day 1, 22 and response evaluation period after neoadjuvant therapy; patients in chemotherapy group complete the evaluation on day 1 of first and second cycle and response evaluation period after neoadjuvant therapy.

FACT-L questionnaire and LCSS scale evaluation in post-operation: patients in tarceva group complete the evaluation on day 1, 22 and 43. Patients in chemotherapy group complete the evaluation on day 1 of first and second cycle and the day after completion of cycle 2 (day 43). Then patients undergo assessment at the time of every 3 months imaging scan (calculating from the first day of adjuvant therapy), until 1 year after adjuvant therapy (calculating from the first day of adjuvant therapy), tumor recurrence, lost follow-up or death (which comes first).

## 3.7.3 Safety parameters and Definitions

The safety analysis population included all patients who received at least one dose of the study drug. Patients’ physical examination results, vital signs, adverse events, and laboratory abnormalities will be summarized. Adverse events should be reported and graded by NCI common terminology for adverse events (CTCAE) version 4.0.

## 3.7.4 Evaluation period

The time of study beginning defined as the date of signing the informed consent form.

#### 3.7.4.1 Research visit

Screening visits should be made within 28 days prior randomization after obtaining informed consent form from patient or his legal representative.

Treatment period begins from patients’ first use of study drug to patients withdraw from study (including pre-operative neo-adjuvant period, surgery, post-operative adjuvant period).

Patients entered recurrence follow-up period after surgery. This period should include post-operation patient visits (see Section 3.6.1 for visits), patients will be follow-up until recurrence or death. If patients recurred after surgery or progressed after neoadjuvant therapy and did not undergo surgery, then they will enter survival follow-up every 3 months until 5 years after surgery or death. If patients progressed after neoadjuvant therapy and unable to undergo surgery, they will receive survival follow-up by telephone every 3 months until 5 years after randomization or death. The follow-up included survival status, time to relapse, the date of death and the cause of death if patients died, and other anti-tumor treatment after relapse.

Patients who progressed in neoadjuvant therapy period will enter into survival follow up every 3 months, until 5 years after randomization or death.

#### 3.7.4.2 Tumor and Response Evaluations

Screening period：Patients must perform thoracic enhanced CT scans (or PET/CT), abdominal enhanced CT scans, cranial-enhanced MRI, and bone scans within 28 days prior to enrollment, and tumor measurements should be recorded in the CRF according to RECIST 1.1 criteria.

Neoadjuvant treatment period: Patients must perform thoracic enhanced CT scans for evaluation of target lesion within 1 week (the 7^th^ week, day 43 to day 49) after 6 weeks of erlotinib and 2 cycles of gemcitabine plus cisplatin, and tumor measurements should be recorded in the CRF according to RECIST 1.1 criteria. In addition, patients should also reassess abdominal enhanced CT scans and cranial-enhanced MRI, ensuring patients did not have metastasis after neoadjuvant therapy. If patients underwent PET/CT in the screening period for evaluation, then response evaluation should also be performed by PET/CT after neoadjuvant therapy.

Visit and follow-up after recurrence: If patients received adjuvant treatment after surgery, a chest-enhanced CT examination should be performed within 28 days before the start of the adjuvant treatment. Postoperative follow-up visits were performed every 3 months ± 5 days from the first day of adjuvant therapy until disease recurrence. Patients should perform thoracic-enhanced CT and abdominal B-ultrasound, cranial-enhanced MRI every 6 months ± 5 days, bone scan every 12 months ± 5 days; and record the status of local relapse or metastasis in the CRF. If patients did not receive adjuvant treatment after surgery, a chest-enhanced CT examination should be performed within 8 weeks. After that, patients would be follow-up every 3 months until disease recurrence. Patients should perform thoracic-enhanced CT and abdominal B-ultrasound, cranial-enhanced MRI every 6 months ± 5 days, bone scan every 12 months ± 5 days; and record the status of local relapse or metastasis in the CRF. Other imaging exam were performed based on symptom or follow-up routines at each research center.

#### 3.7.4.3 Observation and measurement

Patients enrolled in this study will be evaluated by study schedule of activities.

## 3.8 Study process

## 3.8.1 Screening period and baseline

Screening period——within 28 days before administration of study drug

- - - Sign an informed consent form prior to any study-related operations;
    - 12-lead electrocardiogram (ECG) examination at baseline, checked and signed by the investigator;
    - Tumor evaluation at baseline: thoracic-enhanced CT scan or PET/CT should be performed within 28 days before randomization.
    - Cranial-enhanced MRI to exclude brain metastasis, abdominal enhanced CT or B-ultrasound to exclude intra-abdominal metastasis, and bone scan to exclude bone metastasis. If patients have underdone PET/CT scan, they also need to have a cranical enhanced MRI (abdominal enhanced CT and bone scan can be ommitted).
    - Pulmonary function test.
    - Physical examination, including detailed systemic checks. Record the height at the same time.
    - Tumor tissue EGFR test.

Baseline period—within 7 days prior to initiation of study drug

- - - Record/confirm clinical stage and TNM stage at diagnosi.
    - Demographic information, complete medical history, surgical history and smoking history.
    - Vital signs (including heart rate, blood pressure, respiratory rate and body temperature).
    - Record all concomitant disease and medications and their indications.
    - Blood tests: hemoglobin, hematocrit, platelet count, white blood cell count and classification, including the absolute number of neutrophils and lymphocytes.
    - Blood biochemical tests: including blood glucose, calcium, phosphorus, sodium, potassium, chlorine, creatinine, blood urea nitrogen (BUN), total protein, albumin, alanine aminotransferase (ALT), aspartate aminotransferase (AST), alkaline phosphatase , total bilirubin, etc.
    - Perform urine pregnancy test on all women of childbearing age. Postmenopausal women who have had menopause for at least 1 year and women who have undergone sterilization were not required for urine pregnancy test.
    - Urine routine examination: including urine specific gravity, pH, urine sugar, urine protein and occult blood.

## 3.8.2 Neoadjuvant treatment period and surgery period

**Erlotinib group:** Erlotinib administered orally for 6 weeks (42 days) in neoadjuvant treatment period. Response evaluation performed at the 7^th^ week (day 43 to 49). Patients who benefit from neoadjuvant treatment and are resectable judeged by the investigator would undergo surgery at the 8^th^ (day 50 to 56) or 9^th^ (day 57 to 63). Labtatory test, imaging evaluation and quality of life assessment during treatment period are showed in schedule of activities.

**Chemotherapy group:** Patients undergo response evaluation after 2 cycles of gemcitabine plus cisplatin neoadjuvant therapy (the 7^th^ week, day 43 to 49). Patients who benefit from neoadjuvant treatment and are resectable judeged by the investigator would undergo surgery at the 8^th^ (day 50 to 56) or 9^th^ (day 57 to 63). Labtatory test, imaging evaluation and quality of life assessment during treatment period are showed in schedule of activities.

At least 6 stations of lymph nodes should be removed during surgery: 3 stations of N2 lymph nodes (including subcarinal lymph nodes) and 3 stations of N1 lymph nodes.

## 3.8.3 Post-operation adjuvant treatment period

**Erlotinib group:** Erlotinib administered orally for 1 year.

**Chemotherapy group:** gemcitabine plus cisplatin administeredfor 2 cycles.

It is recommended that all surgical patients receive postoperative adjuvant therapy within 8 weeks after surgery (erlotinib group receive erlotinib for 1 year, chemotherapy group receive 2 cycles of gemicitabine and cisplatin). Patients who achieve benefit from neoadjuvant therapy (CR, OR, SD without enlarge and enlarged SD with newly emerged tumor necrosis) will receive the adjuvant therapy showed above. If patients achieve enlarged SD without newly emerged tumor necrosis and undergo surgery, or achieve PD after neoadjuvant therapy and undergo surgery (i.e., satellite lesions appear in ipsilateral thoracic cavity or the same lung lobe), the investigator will determine the adjuvant treatment strategy.

The visit and follow up of chemotherapy group during adjuvant treatment period is similar to that in pre-operation neoadjuvant treatment period (see schedule of activities). The process of blood routine and chemical routine test of erlotinib group in the first 2 cycles (every 21 days) during adjuvant treatment period is similar to that in pre-operation neoadjuvant treatment period；after that, patients undergo blood routine, blood biochemistry, genral condition, thoracic enhanced CT, abdominal B ultrasound at the time of follow-up, namely every 3 months (calculating from the first day of adjuvant treatment) until the end of adjuvant therapy. Patients in the two groups should perform cranial enhanced MRI every 6 months, bone scan every 12 months, until disease recurrence (see schedule of activities). The time, schedule, dose, and duration of postoperative chemotherapy and the time, dose of radiotherapy should be recorded in detail. Blood sample collected at each follow up for exploratory biomarker study.

## Recurrence visit follow-up

Patients entered into recurrence visit follow-up after the end of adjuvant therapy: patients will be followed up every 3 months, including general conditions, symptoms and signs, blood routine, biochemical routine examination, thoracic-enhanced CT and abdominal B-ultrasound (including liver, gallbladder, spleen, pancreas, kidneys, and bilateral adrenal glands); cranial enhanced MRI will be performed every 6 months; bone scan every 12 months. Blood samples will be collected every 3 months within 2 years after surgery for exploratory biomarker analysis; if recurrence occurred 2 years after surgery, blood samples should also be collected at the time of recurrence.

## 3.8.5 Survival follow-up

Patients who had no indication of surgery after neoadjuvant therapy: entered into survival follow-up phase every 3 months until 5 years after randomization.

Patients undergone surgery after neoadjuvant therapy: entered into survival follow-up after recurrence, for obtaining information on overall survival and subsequent treatment after study. Patients will be followed up until 5 years after surgery or or death. The following information should be obtained during each follow-up:

- - - Patients’ survival status.
    - Record date and reason of death if patients died.
    - Disease status, and record the date of recurrence if patients recurred after surgery (for patients who have not relapse in the previous follow-up).
    - Record subsequent anti-cancer treatment after study (including second line and after second line anti-cancer treatment after progression in neoadjuvant treatment, resurgery, anti-cancer treatment after recurrence).

Note: Patients who have relapsed will be judged by clinicians and receive treatment according to routine practice in local center.

## 3.8.6 Temporary visit

Temporary visits should be made according to clinical needs. Clinically significant laboratory abnormalities and adverse events should be recorded in the CRF and original data. If tests repeated in multiple times on the same day, just record the latest testing value in the CRF. However, outliers in all repeated laboratory tests should be recorded in the CRF.

## 3.9 Quality of data

In order to follow the guidelines of the clinical trial quality management practices (GCP), auditors will visit each center regularly, aiming to ensure compliance with research protocols, GCPs and related laws. The interview will include the completeness and clarity of the on-site inspection case report form (CRF), cross-checking the case report form (CRF) and original document, and resolving data queries.

## 3.10 Archive

The information entered in the case report form (CRF) must match the original document. All research document and original materials shoule be retained 15 years after study completion, until receiving written destruction notice from the sponsor.

# 4 Ethical and consideration

## 4.1 Independent ethics committee (IEC)

According to the requirements of the GCP, laws and regulations, and requirements of relevant organizations, all participating research centers should obtain approval documents from the corresponding ethics committee before the study begins. If necessary, it must be amended or re-examined by the ethics committee and forwarded to the investigator.

## 4.2 Ethical guidance for this study

The procedures, evaluation, and documentation involved in this research protocol are intended to ensure that researchers follow the guidelines for clinical practice and the guidelines detailed in the Helsinki Declaration. The implementation of this study will also follow China's corresponding "Quality Management Standards for Clinical Trials"

The investigator cannot modify the research protocol without written consent from ethics committee and sponsor. However, in order to remove risk factors for the patient in emergency, the researcher may deviate or change the study protocol without consent/support from ethics committee/institutional review board/sponsor. The deviations or changes made and reasons should be submitted to Ethics Committee/Institutional Review Board/sponsor as soon as possible, if appropriate, suggestions of research protocol change should also be submitted. The investigator must explain and elucidate all deviations or changes of the research protocol comprehensively.

## 4.3 Patient notice and informed consent

Patients should be provided with the main information and informed consent of the study. Before the study initiation, the investigator must provide patients with informed consent and all other written information approved by the ethics committee. The ethics committee approval and approved patient instructions/informed consent form must be filed together in research documents.

Signed informed consent must be obtained before any specific steps in this study subjected to patients.

## 4.4 Confidentiality

All records relating to patient's identity should be kept confidential and will not open as permitted by applicable laws and/or regulations. Only those who are related to the trial, such as investigators and research nurses, can obtain patient's identity information.

Patient's name does not written in the Case Report Form (CRF). The Case Report Form (CRF) only record patient’s number and name abbreviation. If patient's name shows in any other document (such as a pathology report), a copy of the file must remove the name. Research reports stored in computer must comply with local laws regarding data protection. When the study results are published, patient's identity will also be kept confidential.

The investigator should keep a list to identify patient’s record.

## 4.5 Conditions for protocol amendment

Only after consultation between the sponsor and the principal investigator, modifications can be made to the ongoing trial. The materials prepared by the sponsor for revision shall be reviewed by the principal investigator, biostatisticians and other relevant personnel in advance. Unless emergency adjustments for eliminating harm to patients, or just change in logistical and management aspects of the trial, such as the auditor and telephone number replacement, all program revisions must be submitted to relevant ethics committee and obtained permission. If necessary, it must be submitted to the administration authority. The investigator can only implement the adjustment after the above unit has approved.

## 4.6 Conditions for termination of study

The sponsor and principal investigator reserve the right to terminate the study at any time. If necessary, the parties will arrange the procedures for each study after review and negotiation. When the study is terminated, the sponsor and the principal investigator should ensure that patient's benefit is fully taken into account.

## 4.7 Preservation of research documents, medical report forms and records

## 4.7.1 Preservation of investigators’ documents

In order to ensure that the implementation of the study is fully documented and the research data can then be validated, the investigator must retain a comprehensive and accurate record of the research implementation. These documents should be divided into two different categories: (1) research documentation of the investigator, (2) raw clinical records of patients.

The investigator's research documents include trial protocols and revisions, approval documents from independent ethics committees and government, informed consent samples, drug-related records, personnel resumes, and other related documents/letters.

Patient's raw clinical record (usually pre-defined before the start of the project, recorded outside of the case report form, critical efficacy/safety parameters), usually included patient’s inpatient/outpatient records, records of doctor and nurse, appointments, originals laboratory reports, electrocardiograms, imaging reports, pathology and specialized assessment reports, signed informed consent, consultation letters, patient screening and recruitment forms. Investigators must maintain both types of files for at least 15 years after the study completion or discontinuation. These materials can then be destroyed in accordance with local regulations. If the investigators intend to transfer these research records to other organizations or transport them to other areas, they should inform Roche in advance.

If the investigator cannot guarantee the archiving requirements for any or all documents in the research center, the investigator and the sponsor will make special arrangements to seal the information outside the research center, so that the sealed documents can be returned to the investigator in the circumstance of legal inspection. If patient's follow-up treatment requires the original record, then a copy should be kept outside the research center.

## 4.7.2 Raw record and background data information

Investigators should provide any background data information that the sponsor needed from the research documents and clinical record data as required, especially when it is suspected that errors occurred in the transcription of the data. In the circumstance of special problems and/or government challenge or request for an audit inspection, complete research record should be provided, while patient's privacy should be guaranteed at the same time.

## 4.7.3 Audit and inspection

Researchers should be aware that, upon formal notification, the original records of the study should be prepared and provided to appropriate qualified personnel or their designated personnel, or inspectors from the department of health. The verification of the data in the case report form must be directly checked according to the original record.

## 4.7.4 Case report form

This study used electronic case report form. For each enrolled patient, the Case Report Form (CRF) must be completed and signed by the principal investigator or an authorized representative of the investigator. This condition also applied to CRF records of patients who fail to complete the trial (if a case report form has been completed in the pre-randomization screening period). If patients withdraw from the treatment study, reasons must be recorded on the CRF. If patients withdraw from the study for treatment-related adverse event, try best effort to record the results clearly. The investigator should ensure the accuracy, completeness, legibility, and timeliness of the data recorded in the CRF and all required reports that reported to the sponsor.

# 5 Research audit

If patient’s confidentiality meets local requirements, responsible auditor (or designated person) will contact and visit the investigator regularly, and be allowed to check all test records (case report form and other relevant data) as required.

Throughout the study period, the auditor was responsible for reviewing the case report form regularly, verifying compliance to the study protocol, and checking the completeness, consistency, and accuracy of the input data. The auditor should have access to the laboratory test report and other patient records to verify the input on the case report form. The investigator (or its designee) agrees to cooperate with the auditor to ensure that any issues identified during these audit visits are resolved.

# 6 Publication of research data and protection of trade secrets

Results of the study may be published in journal or at a scientific conference. If this is predictable, the investigator should promise to submit all manuscripts or abstracts to the sponsor in advance of submission, which will enable the sponsor to protect proprietary information. It also helps the sponsor to provide advice based on information from other studies that researchers cannot obtain. Based on standard publications and ethical norms, research data of a multi-center study is supported to be published overall rather than in way of single center data. In this case, a coordinating investigator will be appointed after mutual agreement.

# 7 Statistics and analysis plan

# 7.1 Primary and secondary variables

# 7.1.1 Primary variable

The primary efficacy parameter is objective response rate (ORR).

Objective response rate (ORR) is determined separately for all patients according to the RECIST criteria.

# 7.1.2 Secondary variables

Secondary variables include: downsating rates of pathological lymph nodes, complete resection rate, pCR, PFS, 3 year and 5 year OS rate, quality of life (QoL) and safety data.

Downsating rates of pathological lymph nodes defined as the proportion of patients with pathological confirmed lymph nodes downstaging from N2 to N1 or N0 in the intention to treat (ITT) population.

Pathological complete response (pCR) rate is determined separately for all patients according to the RECIST criteria and pathological criteria.

Complete resection rate defined as the proportion of patients who received completely resection (R0 section) in the intention to treat (ITT) population.

Progression free survival (PFS) defined as the time from randomization to the first confirmed disease progression, relapse or death from any cause, whichever occurs first. For patients underwent surgery, if patient had no disease recurrence or death at the end of the study (or data cut-off), or patients who were lost after surgery were truncated on the date of final confirmed recurrence-free. In patients who did not undergo surgery, the tumor assessment after the end of neoadjuvant therapy was considered as an event for patients with disease progression; patient without disease progression was censored at the time of disease progression after neoadjuvant therapy. Patients who had not undergone a tumor assessment after baseline, the time of randomization was considered as cut-off date.

Progression free survival (PFS) defined as the time from surgery to the first confirmed disease progression or death from any cause, whichever occurs first.

Overall survival (OS) defined as the time from randomization to death from any cause, whichever occurs first. For patients who survived at analysis, their last contact data will be used as cut-off data. The expected survival curve of treatment groups was calculated by Kaplan-Meier method.

Quality of life (QoL) will be assessed by function assessment of cancer therapy - lung (FACT-L) and lung cancer symptom scale (LCSS) for all patients at neoadjuvant treatment phase and adjuvant treatment phase (1 year, calculating from the first day of adjuvant therapy).

# 7.2 Statistics analysis

# 7.2.1 Baseline and demographic characteristics

Baseline, demographic characteristics, baseline tumor characteristics, medical history, concomitant medication, vital signs, and trial termination were included for all enrolled patients. For continuous variables, the mean, standard deviation, distribution range, and median will be calculated; absolute values, frequencies, and percentages will also be calculated.

# 7.2.2 Primary efficacy

The main efficacy variable of this study was objective response rate (ORR), which was defined as the proportion of patients with complete response (CR, Complete Response) and partial response (PR, Partial Response) according to the RECIST 1.1 criteria. Descriptive analysis was used to describe the number and proportion of response categories in each treatment group. At a significance level of 0.05, the difference in overall response rates between each treatment group will be compared using Chi-squared test.

The overall response rate will be analyzed by logistic regression. Logistic regression model will include stratified factors at the time of randomization. Prognostic factors for the interaction analysis between treatment groups were tested for each covariate, and estimates of treatment effects were presented in the form of odds ratios and their 95% two-sided confidence intervals, in order to assess whether the treatment effects of all factor levels were consistent.

# 7.2.3 Secondary efficacy

Variables of secondary efficacy analysis includes:

- Downsating rates of pathological lymph nodes
- Complete resection rate
- Pathological complete response (pCR) rate
- Progression-free survival (PFS)
- 3-year OS rate and 5-year OS rate
- Quality of life (QoL)
- Saftey indicators

# 7.2.4 Exploratory analysis:

- Post-operative 24 week, 48 week and 3 year disease free survival (DFS) rate;
- Explotary biomarker analysis. Potentinal biomarkers include: T790M、cMET、KRAS、ALK-EML4、BRAF、PTEN.

Definication, Chi-squared test will be used to compare the difference of the above 5 rates. The 95% two-sided confidence interval of the difference in the above 3 rates will be calculated using the Anderson-Hauck method.

Survival rate comparsion of progression free survival (PFS), post-operative 24 week, 48 week and 3 year disease free survival (DFS) rate, 3 year and 5 year survival rate will be analyzed by Kaplan-Meier method. Kaplan-Meier test will provide the time of 25th, 75th, and median events and their 95% two-sided confidence interval, and the time to data censoring in the different treatment groups was plotted to investigate differences in follow-up time. The subgroup analyses for progression-free survival by age, gender, smoker, N2 pattem and EGFR mutation status will be analyzed by using a Cox proportional-hazards regression model .

# 7.3 Hypothesis Testing

**7.3.1** The difference in objective response rate will be compared by Chi-squared test. Significance level is 0.05. Establis test hypotheses:

H0：“objective response rate was similar in two treatment groups” versus

H1：“objective response rate was different in two treatment groups”

**7.3.2** The difference in disease-free rate will be compared by Chi-squared test. Significance level is 0.05. Establis test hypotheses:

H0：“disease-free rate was similar in two treatment groups” versus

H1：“disease-free rate was different in two treatment groups”

**7.3.3** The difference in time to event will be compared by two-sided log rank test. Significance level is 0.05. Establis test hypotheses:

H0：“survival function was similar in two treatment groups” versus

H1：“survival function was different in two treatment groups”

# 7.4 Analysis type

Intention to treat population

Statistical analysis will be performed in intend to treat population (ITT), ITT population is defined as all randomized patients.

Safety population

All patients who received at least one dose of study drug will be included in the safety population. Saftey parameters will be analyzed and introduce by the treatment.

# 7.5 Interim analysis

This study does not have an interim analysis plan.

# 7.6 Saftey analysis

For all patients included in safety population, saftey parameters will be analyzed and introduce by the treatment.

- Adverse events
- Serious adverse events
- Laboratory parameters
- Vital signs, including ECOG PS

Adverse events and serious adverse events will be summarized according to NCI CTC-AE version 4, and adverse events will also be summarized according to the severity of events and its relationship with study drug. A descriptive summary of laboratory test values is primarily for outliers. Laboratory abnormalities will also be summarized according to the most severe levels in NCI CTC-AE version 4.0.

# 7.7 Determination of sample size

This study intended to enroll 72 patients, 36 patients in each group, and planned recuriment period is 18 months. According to the principle of substitution, randomized patients cannot be replaced.

The endpoint of this study is to compare the objective response rate (ORR) of erlotinib and gemcitabine plus cisplatin in IIIA-N2 NSCLC with EGFR sensitive mutation. Based on the results of Ch.E.S (Scagliotti GV, et al. J Clin Oncol 26:7508-7508, 2008) and Preoperative chemotherapy plus surgery versus surgery plus adjuvant chemotherapy versus surgery alone in early-stage non-small-cell lung cancer (Felip E, et al. J Clin Oncol 28:3138-45, 2010), the ORR of gemcitabine plus cisplatin induction therapy is about 36%, and the ORR of EGFR TKI in EGFR mutant patients is 70%. With a two-sided significance level of 0.05, this study has 80% (1-β=0.8) power to test difference between the two arms. Assuming a 10% rate of loss to follow-up, a sample size of 72 patients calculating by two independent sample rates comparison would achieve sufficient statistical power to detect a difference between the two arms (patients 1:1 randomized to erlotinib group and gemcitabine plus cisplatin group).

Based on minimization algorithm by Pocock and Simon (1975), patietns will be 1:1 randomized to two groups, and stratified by the following factors: disease status at the beginning of study (single station N2, multi-station N2); gender (male, female); histology (adenocarcinoma, non-adenocarcinoma); smoking status (never vs former vs current). Current smoker means smoking > 100 in a lifetime, and current smoking or smoking cessation time less than 1 year. Past smoker means smoking > 100 in a lifetime, and smoking cessation time less than 1 year. Never smoker means never smoking or smoking < 100 in a lifetime.

# 8. Safety principle

## 8.1 Precautions/ warnings

**Erlotinib**

Erlotinib has been listed at home and abroad, and there has been safety data from large samples.

Common adverse drug reactions (incidence >10%) of erlotinib are: rash, diarrhea, fatigue, shortness of breath, etc. Rare adverse reactions include paronychia, hair and mane abnormalities, and blood biochemical abnormalities.

The warnings and precautions of erlotinib are as follows.

## 8.1.1 Interstitial lung disease (ILD)-like adverse events

Interstitial lung disease (ILD)-like adverse events (including death) are uncommon in the treatment of NSCLC, pancreatic cancer, or other advanced solid tumors are uncommon. In the key study BR 21, the incidence of severe ILD-like adverse events was 0.8% in the placebo and erlotinib groups for NSCLC patients. In the combination study with gemcitabine in pancreatic cancer, the incidence of ILD-like adverse events was 2.5% and 0.4% in the erlotinib plus gemcitabine group and placebo plus gemcitabine group, respectively. In all clinical studies (including non-controlled clinical studies and clinical trials of combination chemotherapy), the overall incidence of erlotinib-treated patients was approximately 0.6%. For example, in patients suspected of ILD-like adverse event, the reported diagnoses include pneumonia, radiation pneumonia, hypersensitivity pneumonitis, interstitial pneumonia, interstitial lung disease, obliterative bronchiolitis, pulmonary fibrosis, acute Respiratory distress syndrome, pulmonary infiltration, and alveolitis. These ILD-like adverse events occur within a few days to a few months after erlotinib treatment. Most of these events are related to confounding factors or influencing factors, such as combination chemotherapy or previous chemotherapy, previous radiotherapy, pre-existing substantial lung disease, metastatic lung disease or lung infection.

If the patient has acute, new or progressive, unexplained lung symptoms, such as dyspnea, coughing and fever, etc., erlotinib should be discontinued pending a diagnostic evaluation. If ILD has been diagnosed, discontinue erlotinib, receive appropriate treatment if necessary(see erlotinib instruction).

## 8.1.2 Diarrhea, dehydration, electrolyte imbalance and kidney failure

Patients developed diarrhea during erlotinib treatment and should be treated with loperamide for moderate or severe diarrhea. In some cases, it may be necessary to reduce the dose. In the case of severe or persistent diarrhea, nausea, loss of appetite, or symptoms of vomiting associated with dehydration, erlotinib should be discontinued and appropriate measures taken to treat dehydration symptoms. Reports of hypokalemia and renal failure, including death, are rare. Some renal failure reports secondary to severe dehydration symptoms caused by diarrhea, vomiting, and/or loss of appetite, while other renal failure reports are confused with combination chemotherapy. Erlotinib should be discontinued in patients with more severe or persistent diarrhea, or symptoms of dehydration, especially in patients with a risk factor (combined medication, symptoms or disease, or other predispositions such as old age). In addition, patients should take appropriate measures and extensive rehydration therapy by intravenous administration. In addition, for patients with a risk of dehydration, renal function and serum electrolytes such as potassium should be monitored (see erlotinib instruction).

## 8.1.3 Hepatitis, liver failure

Rare hepatic failure can occur in erlotinib treatment. The risk of hepatic toxicity is increased in patients with baseline hepatic impairment or use hepatotoxic drugs concomitantly. Therefore, in such patients, regular liver function tests should be considered. Discontinue erlotinib in patients whose has severe abnormal liver tests (see erlotinib instruction).

## 8.1.4 Gastrointestinal perforation

Erlotinib can increase the risk of gastrointestinal perforation, but it is uncommon. If patients receive anti-angiogenesis drugs, corticosteroids, non-steroidal anti-inflammatory drugs (NSAIDs), and/ or taxane-based chemotherapy, or have prior history of peptic ulceration or diverticular disease, may be at increased risk of perforation. Permanently discontinue erlotinib in patients who develop gastrointestinal perforation (see erlotinib instruction).

## 8.1.5 Bullous and exfoliative skin discorders

Bullous, blistering and exfoliative skin conditions, including cases suggestive of Stevens-Johnson syndrome/toxic epidermal necrolysis, which in some cases were fatal, can occur with erlotinib treatment. Interrupt or discontinue Tarceva treatment if the patient develops severe bullous, blistering or exfoliating conditions (see erlotinib instruction).

### 8.1.6 Ocular disorders

Very rare cases of corneal perforation or ulceration were reported during erlotinib administration. Other ocular disorders were reported, including abnormal eyelash growth, keratoconjunctivitis sicca or keratitis, these symptoms are risk factors of corneal perforation or ulceration. Interrupt or discontinue erlotinib therapy if patients present with acute or worsening ocular disorders such as eye pain (see erlotinib instruction).

### 8.1.7 Toxicity caused by interaction between drugs

Erlotinib may cause clinical significant interactions between drugs (see erlotinib instruction).

**Gemcitabine and cisplatin**

For the potential adverse events of gemcitabine and cisplatin, please refer to the detailed prescription information in locally approved packaging for thest two drugs.

## 8.2 Adverse events

## 8.2.1 Definition of adverse events

Defined as an unexpected medical problem that happens during treatment with a drug or other therapy. Adverse event does not necessarily have a causal relationship with this treatment. An AE can therefore be any unfavorable and unintended sign (including an abnormal laboratory finding), symptom, or disease temporally associated with the use of investigational product, whether or not considered related to the investigational product. Worsening of a pre-existing medical condition can be also reported as AE.

Adverse events in the human body (whether or not related to drugs) include the following:

- - - Adverse events occured during the use of medicines by professionals;
    - Adverse events caused by drug overdose (whether intentional or not);
    - Adverse events caused by drug abuse;
    - Adverse events caused by drug discontinuation;
    - Adverse events may be caused solely by patient’s participation in the study (eg, adverse events or serious adverse events caused by discontinuation of anti-hypertensive drugs during the washout period), even if they are unrelated to the study drug.

**No clinical pharmacological effect was observed or achieved will not be considered as adverse events.**

## 8.2.2 Grade of adverse events

Adverse events will be graded by Nation Cancer Institute (NCI) common terminology criteria 4.0 (CTC-AE 4.0), using 5-point scale (grade 1-5) to grade the extent of all adverse events, and record in the CRF in detail. Adverse events ≥3 grade will be futher analyzed.

For **AE not included in CTC-AE**, should be graded as follows:

| **CTC grade** | **Equals to:** | **Definition** |
| --- | --- | --- |
| Grade 1 | Mild | Disconfort, but does not interfere with normal daily activities. |
| Grade 2 | Moderate | Disconfort, and sufficient to reduce or affect daily activities; no indication for treatment or medical intervention, although those measures may improve patients’ overall health or symptoms. |
| Grade 3 | Severe | Incapable of working or performing normal daily activities; treatment or medical interventions is required to improve patients’ overall health or symptoms; delaying the onset of treatment does not directly harm patients’ survival. |
| Grade 4 | Life-threatening/causing disability | Directly threateninglife or causing permanent mental or physical illness, hence hinders work or normal daily activities: treatment or medical intervention is needed to sustain life. |
| Grade 5 | death | AE leading to death |

## 8.2.3 Monitoring of adverse events

Adverse events must be closely monitored, including clinical laboratory testing. Assessment should be performed based on the severity, extent of severity, and relationship to the study drug.

The investigator is responsible for assessing the relationship between all adverse events and study drug. However, the principal investigator can entrust other investigators who participate in the study to judge, but he/she still need to be responsible for it.

## 8.2.4 Relationship between adverse events and study drug

The evaluation of the relationship between an adverse event and study drug is a comprehensive clinical judgment based on all the information obtained in completing the CRF.

Situations assessed as “unrelated” may include:

- - 1. There are clear explanations, such as traumatic bleeding at surgical site;

**OR**

- - 1. Unreasonable, such as a patient hit by a car, but there is no indication that drug-related disorientation caused such event; or cancer occurred only a few days after dosing.

An assessment of “related” indicated that there is reasonable reasons that can explain the adverse event may be related to the study drug.

Factors needed to be considered when assessing the relationship between adverse events and study drug:

- Occurred short-term after drug administration: This adverse event occurred after administration. The clinical evaluation of the event should consider the length of time between the administration of drug and the occurrence of the event.
- The event disappears after stopping the administration (stop stimulation), and the event occurs again after re-dosing (repetitive stimulation): the clinical course of a suspicious event should be fully considered, such as patient's response after stopping the drug (stopping stimulation) or patient's reaction after re-medication (repetitive stimulation).
- Underlying disease, concomitant disease and interstitial disease: the natural course of the disease, the course of treatment, and all other diseases of the patient should be assessed in each report.
- Combined medication or treatment: other medications taken by the patient or other treatments received by the patient should be examined to determine if one of them may cause the adverse event.
- Known response patterns for a certain class of drugs: clinical/preclinical
- Pharmacology and pharmacokinetics of the test drug: The pharmacokinetic characteristics (absorption, distribution, metabolism, and excretion) of the test drug should be considered in conjunction with the individual pharmacodynamic response of each patient.

## 8.2.5 Serious adverse event (SAE):

A serious adverse event is any untoward medical occurance that:

- - - results in death;
    - is life-theratening;
    - requires inpatient hospitalization or causes prolongation of existing hospitalization;
    - results in persistent or significant disability or incapacity;
    - is a congenital anomaly/ birth defect;
    - is an important medical event.

**Life-theratening:** “Life-theratening” defined as “severe”, means an event in which the subject was at risk of death at the time of event. It does not refer to an event which hypothetically might have caused death if it were more severe.

**Hospitalizations：**Any adverse event that causes the patient to be hospitalized or extension of hospitalization is considered serious, except for the following circumstances:

- - - a visit to the emergency room or other hospital department lasting <12 hours; or
    - elective surgery (planned prior to signing consent); or
    - hospitalization unrelated to adverse events (such as for the purpose of recuperation)

It should be noted that any invasive treatment performed during hospitalization may meet the criteria for a “major medical event” and may therefore be reported as a serious adverse event based on clinical judgment. Moreover, if local authorities specifically have more stringent definitions, local regulations prevail.

incapacity：means that someone's ability to engage in daily life is seriously impaired.

According to the standard definition, the term sudden death can only be used when the cause of death caused by heart. The terms death and sudden death are significantly different and therefore must not be used interchangeably.

Any clinical adverse events or abnormal laboratory test results that occur during the study meet a serious grade (according to the above definition), regardless of the treatment received by the patient, must be reported to the sponsor, Drug Administration, Ethics Committee and Shanghai Roche Pharmaceutical Co., Ltd, within one working day after the investigator knows the incident.

Severe adverse events associated with study treatment must be collected and reported, no matter how long after the last dose, even if the trial has ended.

Serious adverse events unrelated to study treatment must be collected and reported during the study period or within 28 days after the last dosing.

## 8.2.6 Non-serious adverse events of special interest (AESI)

Adverse events of special interest in this study include diarrhea, rash and interstitial lung disease-like adverse events. AESI whether related or unrelated to study drug, must be recorded in the CRF and reported to the sponsor within one working day after the investigator knows the incident, and reported to Shanghai Roche Pharmaceutical Co., Ltd regularly.

## 8.2.7 Tumor progression

Death caused by disease progression and disease progression were not reported as SAE in this study.

If progression of potential malignancy is clearly consistent with suspected cancer progression as defined by the RECIST 1.1 criteria or other criteria defined by the study protocol, it did not report as an adverse event. Hospitalization simply due to the progression of potential malignancy did not report as a serious adverse event. Progressive clinical symptoms can be reported as adverse events if it cannot be determined whether these symptoms are due to the progression of potential malignancy, or it did meet the expected mode of disease progression.

Some patients may have worse symptoms, in which patient's clinical symptoms have deteriorated significantly, but tumor measurements do not support disease progression. Or the disease progresses dramatically that the investigator may choose no assessment further. In such cases, clinical progression will be determined based on the worsening of symptoms. These conditions for determining clinical progression were rare, as every effort is needed to determine the objective progression of potential malignancy.

If whether an adverse event is related to disease is unsure, report it as an AE or SAE.

Major medical events: Any adverse event that may harm the patient and may require intervention to prevent a more serious condition can be considered a serious adverse event. To determine major medical events, please refer to the "World Health Organization's Adverse Reaction Terminology - Main Glossary". These terms refer to a serious disease state or a description of a serious disease state. The reason of such incidents reported as serious adverse events was that they may be associated with a serious disease state. As compared to other reporting methods, a SAE report can guarantee special attention and facilitate necessary actions.

## 8.2.8 Unexpected adverse event

An adverse event, which varies in nature, intensity or frequency from information on the investigational drug/agent provided in the Investigator’s Brochure, package insert or safety reports. SUSAR (suspected unexpected serious adverse reaction) means drug-related unexpected adverse events.

## 8.2.9 Reporting of adverse events

Adverse events occurred after signature of informed consent form or within 28 days after the completion of last dose should be reported.

Recorded file must be supported by the original material. laboratory abnormalities related to clinical (eg, those that cause patients withdraw from the study early, require treatment or cause significant clinical manifestations, or those clinically relevant issues judged by the investigator) should be reported as adverse events. A detailed description should be made to each event, including the start and end dates, severity, relationship to the study drug, measures taken, and the outcome of the event.

SAE that meet the definition and adverse events of special interests (AESI) occurred from the signature of the informed consent form to within 28 days after the last dose, including laboratory abnormalities, should be immediately (the within 24 hours after investigator knowledge) report to the person specified in the research document. The SAE report form must also be completed within 24 hours after investigator knowledge and sent to the person designated in the study. The SAE related to study drug must be collected and reported, no matter how long it has been since the last dose, even if the trial has ended. Disease progression in this study was not reported as SAE (see Section 8.2.7).

Each SAE/AESI should be followed up to resolve or stabilization, and submit an updated report to the designated person. A simple grade 4 laboratory abnormality (according to CTC-AE version 4.0) is not reported as a serious adverse reaction unless the investigator believes that the abnormality has reached the criteria of SAE (see Section 8.2.5). Laboratory abnormalities that meet CTC-AE version 4.0 grade 4 criteria and occur as a manifestation of disease during the baseline period should not be reported as SAE, especially if these abnormalities are still present and are allowed to be enrolled or not to be excluded. If there is any doubt about whether the abnormality should be reported as a SAE, the investigator can consult a research inspector. CTC-AE grade 4 laboratory abnormalities should be documented on the “Laboratory Data” page and checked regularly by the Medical inspector. If an adverse event cannot be determined whether it is related to disease, report it as an AE or SAE.

## 8.2.10 Treatment and follow up of adverse events

The final result of each AE must be recorded on the CRF. All AE will be followed up according to the following guidelines:

**Related AE**

Continute follow-up until one of the following results:

- - regress or improve to baseline levels.
  - Causality is again assessed as irrelevant.
  - death.
  - Start a new anti-cancer regimen
  - The inverstigator confirmed that no further improvement was expected.
  - No longer collect the clinical or safety, or the database is eventually closed.

**Unrelated severe or life-threatening AE**

Continute follow-up until one of the following results:

- - regress or improve to baseline levels.
  - Severity improved to grade 2.
  - death.
  - Start a new anti-cancer regimen
  - The inverstigator confirmed that no further improvement was expected.
  - No longer collect the clinical or safety, or the database is eventually closed.

**Unrelated grade 1-2 AE**

Continute follow-up until one of the following results:

- - regress or improve to baseline levels.
  - Start a new anti-cancer regimen
  - The inverstigator confirmed that no further improvement was expected.
  - No longer collect the clinical or safety, or the database is eventually closed.

## 8.2.11 Laboratory test abnormalities

Laboratory abnormalities should be recorded in the CRF. Any laboratory abnormalities meet the criteria of SAE should be captured in the CRF and SAE Report Form.

In the AE page of the CRF, any clinically significant laboratory abnormalities related to treatment should be recorded in the form of a single diagnosis result, ie, the results are consistent with one or more of the following:

- - Clinical complications
  - Required change of study drug (such as dose reduction, discontinuation or permanently drug stop).
  - Required change ofcombined treatment regimen (eg. addition, interruption, discontinuation, or any other change to a combination drug, treatment, or medication).

This applies to any protocol and non- protocol defined laboratory safety and efficacy outcomes obtained from an examination performed after the first dose of study drug, those results exceed the laboratory reference and meets clinically significant criteria.

This does not apply to laboratory results that exceed the laboratory reference and meets clinically significant criteria (will be analyzed and reported by laboratory abnormalities); AE that is explicitly excluded by the protocol; or results caused by reported or being reported AE.

If a medically significant, unexplained laboratory test abnormality occurred, the test should be repeated and followed up until it returns to the normal range and/or if it is found that the abnormal condition can be properly interpreted. If it has been clearly explained, it should be recorded on the CRF.

## 8.3 Pregnancy

If a female patient is pregnant during the study, she must stop using study drug as directed and notify the investigator immediately. The investigator must report pregnancy through the clinical trial pregnancy report form to the sponsor, Shanghai Roche Pharmaceutical Co., Ltd., drug regulatory department and ethics committee within 24 hours. The investigator should give the patient medical advice and discuss the risks of continuing pregnancy and the possible effects on the fetus. Patients should be monitored until the end of pregnancy, and if the baby born is alive, the baby should also be followed up. Pregnancy that occurs within 90 days after the completion of study also should be reported to the investigator.

If the spouse of male patient enrolled in the study is pregnant or is pregnant within 90 days after the completion of the study drug, then the informed consent form signed by the pregnant spouse must be obtained through the Pregnancy Spouse Data Release Form, and the pregnant spouse showed be followed up, and report the result of pregnancy to the investigator and sponsor. The spouse should be consulted to discuss the risk of continuing pregnancy and possible adverse effects on the fetus. Patients should continue to be monitored until the end of pregnancy. If the baby born is alive, the baby should also be followed up.

**9 Independent review committee (IRC)**

Independent review committee (IRC) includes two certified radiologists who have been audited to assess the results of radiological examinations and a certified oncologist. They will independently review the patient's imaging images (CT, MRI, PET/CT, bone scan) according to the RECIST 1.1 criteria to confirm response/non-response/progression (CR, PR, SD or PD), or postoperative local recurrence or metastasis.

The review by the Independent review committee does not determine whether a patient is eligible or not for the study, nor does it determine the patient's treatment. The investigator made all treatment decisions based on local assessment results. The main analysis of the objective response rate, PFS, and DFS were based on the results of investigator's assessment.

The radiographic examination (CT, MRI, PET/CT, and bone scan) at baseline, pre-neoadjuvant treatment, and post-operation regular follow-up visits were all required to follow the image acquisition of this study, and all those data should be forwarded to independent reviewers.

# 10. Appendix

## 10.1 General condition (ECOG Performance status)

| **Grade** | **ECOG** |
| --- | --- |
| 0 | Fully active, able to carry on all pre-disease performance without restriction (Karnofsky 90-100) |
| 1 | Restricted in physically strenuous activity but ambulatory and able to carry out work of a light or sedentary nature (e.g., light house work, office work). (Karnofsky 70-80) |
| 2 | Ambulatory and capable of all selfcare but unable to carry out any work activities. Up and about more than 50% of waking hours (Karnofsky 50-60) |
| 3 | Capable of only limited selfcare, confined to bed or chair more than 50% of waking hours. (Karnofsky 30-40) |
| 4 | Completely disabled. Cannot carry on any selfcare. Totally confined to bed or chair. (Karnofsky 10-20) |

## 10.2 Lung cancer international TNM staging classification

**The 7^th^ TNM staging (international union against cancer (UICC) 2009 edition)**

**Primary tumor (T)**

**TX** Primary tumour cannot be assessed, or tumour proven by the presence of malignant cells in sputum or bronchial washings but not visualised by imaging or bronchoscopy.

**T0** No evidence of primary tumour.

**Tis** Carcinoma in situ.

**T1** Tumour 3cm or less in greatest dimension, surrounded by lung or visceral pleura, without bronchoscopic evidence of invasion more proximal than the lobar bronchus (ie not in the main bronchus).

**T1a** Tumour 2cm or less in greatest dimension

**T1b**  Tumour more than 2cm but 3cm or less in greatest dimension

**T2** Tumour more than 3cm but 7cm or less or tumour with any of the following features (T2 tumours with these features are classified T2a if 5cm or less)

**T2a** Tumour more than 3 cm but 5cm or less in greatest dimension

**T2b**  Tumour more than 5 cm but 7cm or less in greatest dimension

**T3** Tumour more than 7cm or one that directly invades any of the following: parietal pleural (PL3) chest wall (including superior sulcus tumours), diaphragm, phrenic nerve, mediastinal pleura, parietal pericardium; or tumour in the main bronchus (less than 2cm distal to the carina*) but without involvement of the carina; or associated atelectasis or obstructive pneumonitis of the entire lung; or separate tumour nodule(s) in the same lobe.

**T4** Tumour of any size that invades any of the following: mediastinum, heart, great vessels, trachea, recurrent laryngeal nerve, oesophagus, vertebral body, carina; separate tumour nodule(s) in a different ipsilateral lobe.

**Regional lymph nodes (N)**

**NX** Regional lymph nodes cannot be assessed

**N0** No regional node metastasis

**N1** Metastasis in ipsilateral peribronchial and/or ipsilateral hilar lymph nodes and intrapulmonary nodes, including involvement by direct extension.

**N2** Metastasis in ipsilateral mediastinal and/or subcarinal lymph node(s)

**N3** Metastasis in contralateral mediastinal, contralateral hilar, ipsilateral or contralateral scalene, or supraclavicular lymph node(s)

**Distant metastasis (M)**

**Mx** metastasis cannot be assessed

**M0** No distant metastasis

**M1** Distant metasis

**M1a**  Separate tumour nodule(s) in a contralateral lobe; tumour with pleural nodules or malignant pleural (or pericardial) effusion

**M1b** Distant metasis (in extrathoracic organs)

**Clinical staging（UICC 2009 edition）**

**Occult carcinoma**  TX N0 M0

**Stage 0** Tis N0 M0

**Stage IA** T1 N0 M0

**Stage IB** T2a N0 M0

**Stage IIA** T1 N1 M0, T2a N1 M0, T2b N0 M0

**Stage II B** T2b N1 M0, T3 N0 M0

**Stage IIIA** T4 N0 M0, T3-4 N1 M0, T1-3 N2 M0

**Stage IIIB** T4 N2 M0, T1-4 N3 M0

**Stage IV** T1-4 N0-3 M1

(The following table summarizes the above staging)

| T/M | N0 | N1 | N2 | N3 |
| --- | --- | --- | --- | --- |
| T1a | IA | IIA | IIIA | IIIB |
| T1b | IA | IIA | IIIA | IIIB |
| T2a | IB | IIA | IIIA | IIIB |
| T2b | IIA | IIB | IIIA | IIIB |
| T3 | IIB | IIIA | IIIA | IIIB |
|  | IIB | IIIA | IIIA | IIIB |
|  | IIB | IIIA | IIIA | IIIB |
| T4 | IIIA | IIIA | IIIB | IIIB |
|  | IIIA | IIIA | IIIB | IIIB |
| M1a | IV | IV | IV | IV |
|  | IV | IV | IV | IV |
| M1b | IV | IV | IV | IV |

## 10.3 RECIST1.1 tumor response evaluation criteria

**Measurablility of tumor at baseline**

**(1.) Definitions**

At baseline, tumor lesions/lymph nodes will be categorised measurable or non-measurable as follows:

**(1.1.) Measurable**

Tumor lesions: Must be accurately measured in at least one dimension (longest diameter in the plane of measurement is to be recorded) with a minimum size of:

• 10mm by CT scan (CT scan slice thickness no greater than 5mm) .

•10mm caliper measurement by clinical exam (lesions which cannot be accurately measured with calipers should be recorded as non-measurable).

• 20mm by chest X-ray.

Malignant lymph nodes: To be considered pathologically enlarged and measurable, a lymph node must be P15mm in short axis when assessed by CT scan (CT scan slice thickness recommended to be no greater than 5 mm). At baseline and in follow-up, only the short axis will be measured and followed.

**(1.2.) Non-measurable**

All other lesions, including small lesions (longest diameter <10mm or pathological lymph nodes with P10 to <15mm short axis) as well as truly non-measurable lesions. Lesions considered truly non-measurable include: leptomeningeal disease, ascites, pleural or pericardial effusion, inflammatory breast disease, lymphangitic involvement of skin or lung, abdominal masses/abdominal organomegaly identified by physical exam that is not measurable by reproducible imaging techniques.

**(1.3.) Special considerations regarding lesion measurability**

Bone lesions, cystic lesions, and lesions previously treated with local therapy require particular comment:

Bone lesions:

• Bone scan, PET scan or plain films are not considered adequate imaging techniques to measure bone lesions. However, these techniques can be used to confirm the presence or disappearance of bone lesions.

• Lytic bone lesions or mixed lytic-blastic lesions, with identifiable soft tissue components, that can be evaluated by cross sectional imaging techniques such as CTor MRI can be considered as measurable lesions if the soft tissue component meets the definition of measurability described above.

• Blastic bone lesions are non-measurable.

Cystic lesions:

• Lesions that meet the criteria for radiographically defined simple cysts should not be considered as malignant lesions (neither measurable nor non-measurable) since they are, by definition, simple cysts.

• ‘Cystic lesions’ thought to represent cystic metastases can be considered as measurable lesions, if they meet the definition of measurability described above. However, if noncystic lesions are present in the same patient, these are preferred for selection as target lesions.

Lesions with prior local treatment:

• Tumour lesions situated in a previously irradiated area, or in an area subjected to other loco-regional therapy, are usually not considered measurable unless there has been demonstrated progression in the lesion. Study protocols should detail the conditions under which such lesions would be considered measurable.

**(2.) Specifications by methods of measurements**

**(2.1.) Measurement of lesions**

All measurements should be recorded in metric notation, using calipers if clinically assessed. All baseline evaluations should be performed as close as possible to the treatment start and never more than 4 weeks before the beginning of the treatment.

**(2.2.) Method of assessment**

The same method of assessment and the same technique should be used to characterise each identified and reported lesion at baseline and during follow-up. Imaging based evaluation should always be done rather than clinical examination unless the lesion(s) being followed cannot be imaged but are assessable by clinical exam.

Clinical lesions: Clinical lesions will only be considered measurable when they are superficial and P10mm diameter as assessed using calipers (e.g. skin nodules). For the case of skin lesions, documentation by colour photography including a ruler to estimate the size of the lesion is suggested. As noted above, when lesions can be evaluated by both clinical exam and imaging, imaging evaluation should be undertaken since it is more objective and may also be reviewed at the end of the study.

Chest X-ray: Chest CT is preferred over chest X-ray, particularly when progression is an important endpoint, since CT is more sensitive than X-ray, particularly in identifying new lesions. However, lesions on chest X-ray may be considered measurable if they are clearly defined and surrounded by aerated lung.

CT, MRI: CT is the best currently available and reproducible method to measure lesions selected for response assessment. This guideline has defined measurability of lesions on CT scan based on the assumption that CT slice thickness is 5mm or less. As is described in Appendix II, when CT scans have slice thickness greater than 5 mm, the minimum size for a measurable lesion should be twice the slice thickness. MRI is also acceptable in certain situations (e.g. for body scans).

Ultrasound: Ultrasound is not useful in assessment of lesion size and should not be used as a method of measurement. Ultrasound examinations cannot be reproduced in their entirety for independent review at a later date and, because they are operator dependent, it cannot be guaranteed that the same technique and measurements will be taken from one assessment to the next. If new lesions are identified by ultrasound in the course of the study, confirmation by CT or MRI is advised. If there is concern about radiation exposure at CT, MRI may be used instead of CT in selected instances.

Endoscopy, laparoscopy: The utilisation of these techniques for objective tumour evaluation is not advised. However, they can be useful to confirm complete pathological response when biopsies are obtained or to determine relapse in trials where recurrence following complete response or surgical resection is an endpoint.

Tumour markers: Tumour markers alone cannot be used to assess objective tumour response. If markers are initially above the upper normal limit, however, they must normalise for a patient to be considered in complete response. Because tumour markers are disease specific, instructions for their measurement should be incorporated into protocols on a disease specific basis.

Cytology, histology: These techniques can be used to differentiate between PR and CR in rare cases if required by protocol (for example, residual lesions in tumour types such as germ cell tumours, where known residual benign tumours can remain). When effusions are known to be a potential adverse effect of treatment (e.g. with certain taxane compounds or angiogenesis inhibitors), the cytological confirmation of the neoplastic origin of any effusion that appears or worsens during treatment can be considered if the measurable tumour has met criteria for response or stable disease in order to differentiate between response (or stable disease) and progressive disease.

**Tumour response evaluation**

**(1.) Assessment of overall tumour burden and measurable disease**

To assess objective response or future progression, it is necessary to estimate the overall tumour burden at baseline and use this as a comparator for subsequent measurements. Only patients with measurable disease at baseline should be included in protocols where objective tumour response is the primary endpoint. Measurable disease is defined by the presence of at least one measurable lesion. In studies where the primary endpoint is tumour progression (either time to progression or proportion with progression at a fixed date), the protocol must specify if entry is restricted to those with measurable disease or whether patients having non-measurable disease only are also eligible.

**(2.) Baseline documentation of ‘target’ and ‘non-target’ lesions**

When more than one measurable lesion is present at baseline all lesions up to a maximum of five lesions total (and a maximum of two lesions per organ) representative of all involved organs should be identified as target lesions and will be recorded and measured at baseline (this means in instances where patients have only one or two organ sites involved a maximum of two and four lesions respectively will be recorded).

Target lesions should be selected on the basis of their size (lesions with the longest diameter), be representative of all involved organs, but in addition should be those that lend themselves to reproducible repeated measurements. It may be the case that, on occasion, the largest lesion does not lend itself to reproducible measurement in which circumstance the next largest lesion which can be measured reproducibly should be selected.

Lymph nodes merit special mention since they are normal anatomical structures which may be visible by imaging even if not involved by tumour. Pathological nodes which are defined as measurable and may be identified as target lesions must meet the criterion of a short axis of >=15mm by CT scan. Only the short axis of these nodes will contribute to the baseline sum. The short axis of the node is the diameter normally used by radiologists to judge if a node is involved by solid tumour. Nodal size is normally reported as two dimensions in the plane in which the image is obtained (for CT scan this is almost always the axial plane; for MRI the plane of acquisition may be axial, saggital or coronal). The smaller of these measures is the short axis. For example, an abdominal node which is reported as being 20mm X 30mm has a short axis of 20mm and qualifies as a malignant, measurable node. In this example, 20mm should be recorded as the node measurement. All other pathological nodes (those with short axis P10mm but <15 mm) should be considered non-target lesions. Nodes that have a short axis <10mm are considered non-pathological and should not be recorded or followed. A sum of the diameters (longest for non-nodal lesions, short axis for nodal lesions) for all target lesions will be calculated and reported as the baseline sum diameters. If lymph nodes are to be included in the sum, then as noted above, only the short axis is added into the sum. The baseline sum diameters will be used as reference to further characterise any objective tumour regression in the measurable dimension of the disease.

All other lesions (or sites of disease) including pathological lymph nodes should be identified as non-target lesions and should also be recorded at baseline. Measurements are not required and these lesions should be followed as ‘present’, ‘absent’, or in rare cases ‘unequivocal progression’ (more details to follow). In addition, it is possible to record multiple nontarget lesions involving the same organ as a single item on the case record form (e.g. ‘multiple enlarged pelvic lymph nodes’ or ‘multiple liver metastases’).

**(3.) Response criteria**

This section provides the definitions of the criteria used to determine objective tumour response for target lesions.

**(3.1.) Evaluation of target lesions**

Complete Response (CR): Disappearance of all target lesions. Any pathological lymph nodes (whether target or non-target) must have reduction in short axis to <10 mm.

Partial Response (PR): At least a 30% decrease in the sum of diameters of target lesions, taking as reference the baseline sum diameters.

Progressive Disease (PD): At least a 20% increase in the sum of diameters of target lesions, taking as reference the smallest sum on study (this includes the baseline sum if that is the smallest on study). In addition to the relative increase of 20%, the sum must also demonstrate an absolute increase of at least 5 mm. (Note: the appearance of one or more new lesions is also considered progression).

Stable Disease (SD): Neither sufficient shrinkage to qualify for PR nor sufficient increase to qualify for PD, taking as reference the smallest sumdiameterswhile on study.

**(3.2.) Special notes on the assessment of target lesions**

Lymph nodes：Lymph nodes identified as target lesions should always have the actual short axis measurement recorded (measured in the same anatomical plane as the baseline examination), even if the nodes regress to below 10mm on study. This means that when lymph nodes are included as target lesions, the ‘sum’ of lesions may not be zero even if complete response criteria aremet, since a normal lymph node is defined as having a short axis of <10mm. Case report forms or other data collection methods may therefore be designed to have target nodal lesions recorded in a separate section where, in order to qualify for CR, each node must achieve a short axis <10mm. For PR, SD and PD, the actual short axis measurement of the nodes is to be included in the sum of target lesions. Target lesions that become ‘too small to measure’. While on study, all lesions (nodal and non-nodal) recorded at baseline should have their actual measurements recorded at each subsequent evaluation, even when very small (e.g. 2mm). However, sometimes lesions or lymph nodes which are recorded as target lesions at baseline become so faint on CT scan that the radiologist may not feel comfortable assigning an exact measure and may report themas being ‘too small to measure’. When this occurs it is important that a value be recorded on the case report form. If it is the opinion of the radiologist that the lesion has likely disappeared, the measurement should be recorded as 0mm. If the lesion is believed to be present and is faintly seen but too small to measure, a default value of 5mm should be assigned (Note: It is less likely that this rule will be used for lymph nodes since they usually have a definable size when normal and are frequently surrounded by fat such as in the retroperitoneum; however, if a lymph node is believed to be present and is faintly seen but too small to measure, a default value of 5mmshould be assigned in this circumstance as well). This default value is derived from the 5mm CT slice thickness (but should not be changed with varying CT slice thickness). The measurement of these lesions is potentially non-reproducible, therefore providing this default value will prevent false responses or progressions based upon measurement error. To reiterate, however, if the radiologist is able to provide an actual measure, that should be recorded, even if it is below 5mm.

Lesions that split or coalesce on treatment. When non-nodal lesions ‘fragment’, the longest diameters of the fragmented portions should be added together to calculate the target lesion sum. Similarly, as lesions coalesce, a plane between them may be maintained that would aid in obtaining maximal diameter measurements of each individual lesion. If the lesions have truly coalesced such that they are no longer separable, the vector of the longest diameter in this instance should be the maximal longest diameter for the ‘coalesced lesion’.

**(3.3.) Evaluation of non-target lesions**

This section provides the definitionsof the criteriausedtodetermine the tumour response for the group of non-target lesions.

While some non-target lesions may actually be measurable, they need not bemeasured and instead should be assessed only qualitatively at the time points specified in the protocol.

Complete Response (CR): Disappearance of all non-target lesions and normalisation of tumour marker level. All lymph nodes must be non-pathological in size (<10mm short axis).

Non-CR/Non-PD: Persistence of one or more non-target lesion(s) and/or maintenance of tumour marker level above the normal limits.

Progressive Disease (PD): Unequivocal progression of existing non-target lesions. (Note: the appearance of one or more new lesions is also considered progression).

**(3.4.) Special notes on assessment of progression of nontarget disease**

The concept of progression of non-target disease requires additional explanation as follows:

When the patient also has measurable disease. In this setting, to achieve ‘unequivocal progression’ on the basis of the non-target disease, there must be an overall level of substantial worsening in non-target disease such that, even in presence of SD or PR in target disease, the overall tumour burden has increased sufficiently to merit discontinuation of therapy. A modest ‘increase’ in the size of one or more non-target lesions is usually not sufficient to quality for unequivocal progression status. The designation of overall progression solely on the basis of change in non-target disease in the face of SD or PR of target disease will therefore be extremely rare.

When the patient has only non-measurable disease. This circumstance arises in some phase III trials when it is not a criterion of study entry to have measurable disease. The same general concepts apply here as noted above, however, in this instance there is no measurable disease assessment to factor into the interpretation of an increase in non-measurable disease burden. Because worsening in non-target disease cannot be easily quantified (by definition: if all lesions are truly non-measurable) a useful test that can be applied when assessing patients for unequivocal progression is to consider if the increase in overall disease burden based on the change in non-measurable disease is comparable in magnitude to the increase that would be required to declare PD for measurable disease: i.e. an increase in tumour burden representing an additional 73% increase in ‘volume’ (which is equivalent to a 20% increase diameter in a measurable lesion). Examples include an increase in a pleural effusion from ‘trace’ to ‘large’, an increase in lymphangitic disease from localised to widespread, or may be described in protocols as ‘sufficient to require a change in therapy’. If ‘unequivocal progression’ is seen, the patient should be considered to have had overall PD at that point. While it would be ideal to have objective criteria to apply to non-measurable disease, the very nature of that disease makes it impossible to do so, therefore the increasemust be substantial.

**(3.5.) New lesions**

The appearance of new malignant lesions denotes disease progression; therefore, some comments on detection of new lesions are important. There are no specific criteria for the identification of new radiographic lesions; however, the finding of a new lesion should be unequivocal: i.e. not attributable to differences in scanning technique, change in imaging modality or findings thought to represent something other than tumour (for example, some ‘new’ bone lesions may be simply healing or flare of pre-existing lesions). This is particularly important when the patient’s baseline lesions show partial or complete response. For example, necrosis of a liver lesion may be reported on a CT scan report as a ‘new’ cystic lesion, which it is not.

A lesion identified on a follow-up study in an anatomical location that was not scanned at baseline is considered a new lesion and will indicate disease progression. An example of this is the patient who has visceral disease at baseline and while on study has a CT or MRI brain ordered which reveals metastases. The patient’s brain metastases are considered to be evidence of PD even if he/she did not have brain imaging at baseline. If a new lesion is equivocal, for example because of its small size, continued therapy and follow-up evaluation will clarify if it represents truly new disease. If repeat scans confirm there is definitely a new lesion, then progression should be declared using the date of the initial scan.

While FDG-PET response assessments need additional study, it is sometimes reasonable to incorporate the use of FDG-PET scanning to complement CT scanning in assessment of progression (particularly possible ‘new’ disease). New lesions on the basis of FDG-PET imaging can be identified according to the following algorithm:

a) Negative FDG-PET at baseline, with a positivel FDG-PET at follow-up is a sign of PD based on a new lesion.

b) No FDG-PET at baseline and a positive FDG-PET at follow- up:

If the positive FDG-PET at follow-up corresponds to a new site of disease confirmed by CT, this is PD.

If the positive FDG-PET at follow-up is not confirmed as a new site of disease on CT, additional follow-up CT scans are needed to determine if there is truly progression occurring at that site (if so, the date of PD will be the date of the initial abnormal FDG-PET scan).

If the positive FDG-PET at follow-up corresponds to a pre-existing site of disease on CT that is not progressing on the basis of the anatomic images, this is not PD.

**(4.) Evaluation of best overall response**

The best overall response is the best response recorded from the start of the study treatment until the end of treatment taking into account any requirement for confirmation. On occasion a response may not be documented until after the end of therapy so protocols should be clear if post-treatment assessments are to be considered in determination of best overall response. Protocols must specify how any new therapy introduced before progression will affect best response designation. The patient’s best overall response assignment will depend on the findings of both target and non-target disease and will also take into consideration the appearance of new lesions. Furthermore, depending on the nature of the study and the protocol requirements, it may also require confirmatory measurement (see Section 4.6). Specifically, in non-randomised trials where response is the primary endpoint, confirmation of PR or CR is needed to deem either one the ‘best overall response’. This is described further below.

**(4.1.) Time point response**

It is assumed that at each protocol specified time point, a response assessment occurs. Table 1 on the next page provides a summary of the overall response status calculation at each time point for patients who have measurable disease at baseline.

When patients have non-measurable (therefore non-target) disease only, Table 1 is to be used.

Table 1 Time point response: patients with target (+/- non-target) disease.

| Target lesions | Non-target lesion | New lesion | Overall response |
| --- | --- | --- | --- |
| CR | CR | No | CR |
| CR | N-onCR/non-PD | No | PR |
| CR | Not evaluated | No | PR |
| PR | Non-PD or not all evaluated | No | PR |
|  | Not all evaluated |  |  |
| SD | Non-PD or not all evaluated | No | SD |
|  |  |  |  |
| Not all evaluated | Non-PD | No | NE |
| PD | Any | Yes or No | PD |
| Any | PD | Yes or No | PD |
| Any | Any | Yes | PD |
| CR = complete response, PR = partial response, SD = stable disease, PD = progressive disease, NE = inevaluable. | | | |

Table 2 Time point response: patients with non-target disease only.

| Non-target lesions | New lesions | Overall response |
| --- | --- | --- |
| CR | No | CR |
| Non-CR/non-PD | No | Non-CR/non-PD^a^ |
| Not all evaluated | No | NE |
| Unequivocal PD | Yes or No | PD |
| Any | Yes | PD |
| CR = complete reponse, PD = progressive disease, NE = inevaluable. | | |
| a “Non-CR/non-PD” is preferred over “stable disease” for non-target disease since SD is increasingly used as endpoint for assessment of efficacy in some trials so to assign this category when no lesions can be measured is not advised. | | |

（4.2.）Missing assessments and inevaluable designation

When no imaging/measurement is done at all at a particular time point, the patient is not evaluable (NE) at that time point. If only a subset of lesion measurements are made at an assessment, usually the case is also considered NE at that time point, unless a convincing argument can be made that the contribution of the individual missing lesion(s) would not change the assigned time point response. This would be most likely to happen in the case of PD. For example, if a patient had a baseline sum of 50mm with three measured lesions and at follow-up only two lesions were assessed, but those gave a sum of 80 mm, the patient will have achieved PD status, regardless of the contribution of the missing lesion.

**（4.3.）Best overall response: all time points**

The best overall response is determined once all the data for the patient is known.

Best response determination in trials where confirmation of complete or partial response IS NOT required: Best response in these trials is defined as the best response across all time points (for example, a patient who has SD at first assessment, PR at second assessment, and PD on last assessment has a best overall response of PR). When SD is believed to be best response, it must also meet the protocol specified minimum time from baseline. If the minimum time is not met when SD is otherwise the best time point response, the patient’s best response depends on the subsequent assessments. For example, a patient who has SD at first assessment, PD at second and does not meet minimum duration for SD, will have a best response of PD. The same patient lost to follow-up after the first SD assessment would be considered inevaluable. Best response determination in trials where confirmation of complete or partial response IS required: Complete or partial responses may be claimed only if the criteria for each are met at a subsequent time point as specified in the protocol (generally 4 weeks later). In this circumstance, the best overall response can be interpreted as in Table 3.

Table 3 Best overall response when confirmation of CR and PR required.

| Overall response | Overall response | Best overall response |
| --- | --- | --- |
| First time point | Subsequent time point |  |
| CR | CR | CR |
| CR | PR | SD、PD or PR^a^ |
| CR | SD | SD provided minimum criteria for SD duration met, otherwise, PD |
| CR | PD | SD provided minimum criteria for SD duration met, otherwise, PD |
| CR | NE | SD provided minimum criteria for SD duration met, otherwise NE |
| PR | CR | PR |
| PR | PR | PR |
| PR | SD | SD |
| PR | PD | SD provided minimum criteria for SD duration met, otherwise, PD |
| PR | NE | SD provided minimum criteria for SD duration met, otherwise NE |
| NE | NE | NE |
| CR = complete response, PR = partial response, SD = stable disease, PD = progressive disease, and NE = inevaluable. | | |
| a If a CR is truly met at first time point, then any disease seen at a subsequent time point, even disease meeting PR criteria relative to baseline, makes the disease PD at that point (since disease must have reappeared after CR). Best response would depend on whether minimum duration for SD was met. However, sometimes ‘CR’ may be claimed when subsequent scans suggest small lesions were likely still present and in fact the patient had PR, not CR at the first time point. Under these circumstances, the original CR should be changed to PR and the best response is PR. | | |

**(4.4.) Special notes on response assessment**

When nodal disease is included in the sum of target lesions and the nodes decrease to ‘normal’ size (<10 mm), they may still have a measurement reported on scans. This measurement should be recorded even though the nodes are normal in order not to overstate progression should it be based on increase in size of the nodes. As noted earlier, this means that patients with CR may not have a total sum of ‘zero’ on the case report form (CRF).

In trials where confirmation of response is required, repeated ‘NE’ time point assessments may complicate best response determination. The analysis plan for the trial must address how missing data/assessments will be addressed in determination of response and progression. For example, in most trials it is reasonable to consider a patient with time point responses of PR-NE-PR as a confirmed response.

Patients with a global deterioration of health status requiring discontinuation of treatment without objective evidence of disease progression at that time should be reported as ‘symptomatic deterioration’. Every effort should be made to document objective progression even after discontinuation of treatment. Symptomatic deterioration is not a descriptor of an objective response: it is a reason for stopping study therapy. The objective response status of such patients is to be determined by evaluation of target and non-target disease as shown in Tables 1–3.

Conditions that define ‘early progression, early death and inevaluability’ are study specific and should be clearly described in each protocol (depending on treatment duration, treatment periodicity).

In some circumstances it may be difficult to distinguish residual disease from normal tissue. When the evaluation of complete response depends upon this determination, it is recommended that the residual lesion be investigated (fine needle aspirate/biopsy) before assigning a status of complete response. FDG-PET may be used to upgrade a response to a CR in a manner similar to a biopsy in cases where a residual radiographic abnormality is thought to represent fibrosis or scarring. The use of FDG-PET in this circumstance should be prospectively described in the protocol and supported by disease specific medical literature for the indication. However, it must be acknowledged that both approaches may lead to false positive CR due to limitations of FDG-PETand biopsy resolution/ sensitivity.

For equivocal findings of progression (e.g. very small and uncertain new lesions; cystic changes or necrosis in existing lesions), treatment may continue until the next scheduled assessment. If at the next scheduled assessment, progression is confirmed, the date of progression should be the earlier date when progression was suspected.

**(4.5.) Frequency of tumor re-evaluation**

Frequency of tumour re-evaluation while on treatment should be protocol specific and adapted to the type and schedule of treatment. However, in the context of phase II studies where the beneficial effect of therapy is not known, follow-up every 6–8 weeks (timed to coincide with the end of a cycle) is reasonable. Smaller or greater time intervals than these could be justified in specific regimens or circumstances. The protocol should specify which organ sites are to be evaluated at baseline (usually those most likely to be involved with metastatic disease for the tumour type under study) and how often evaluations are repeated. Normally, all target and non-target sites are evaluated at each assessment. In selected circumstances certain non-target organs may be evaluated less frequently. For example, bone scans may need to be repeated only when complete response is identified in target disease or when progression in bone is suspected.

After the end of the treatment, the need for repetitive tumour evaluations depends on whether the trial has as a goal the response rate or the time to an event (progression/death). If ‘time to an event’ (e.g. time to progression, disease-free survival, progression-free survival) is the main endpoint of the study, then routine scheduled re-evaluation of protocol specified sites of disease is warranted. In randomised comparative trials in particular, the scheduled assessments should be performed as identified on a calendar schedule (for example: every 6–8 weeks on treatment or every 3–4 months after treatment) and should not be affected by delays in therapy, drug holidays or any other events that might lead to imbalance in a treatment arm in the timing of disease assessment.

**(4.6.) Confirmatory measurement/duration of response**

**(4.6.1.) Confirmatory**

In non-randomised trials where response is the primary endpoint, confirmation of PR and CR is required to ensure responses identified are not the result of measurement error. This will also permit appropriate interpretation of results in the context of historical data where response has traditionally required confirmation in such trials. However, in all other circumstances, i.e. in randomised trials (phase II or III) or studies where stable disease or progression are the primary endpoints, confirmation of response is not required since it will not add value to the interpretation of trial results. However, elimination of the requirement for response confirmation may increase the importance of central review to protect against bias, in particular in studies which are not blinded.

In the case of SD, measurements must have met the SD criteria at least once after study entry at a minimum interval (in general not less than 6–8 weeks) that is defined in the study protocol.

**(4.6.2.) Duration of overall response**

The duration of overall response is measured from the time measurement criteria are first met for CR/PR (whichever is first recorded) until the first date that recurrent or progressive disease is objectively documented (taking as reference for progressive disease the smallest measurements recorded on study).

The duration of overall complete response is measured from the time measurement criteria are first met for CR until the first date that recurrent disease is objectively documented.

**(4.6.3.) Duration of stable disease**

Stable disease is measured from the start of the treatment (in randomised trials, from date of randomisation) until the criteria for progression are met, taking as reference the smallest sum on study (if the baseline sum is the smallest, this is the reference for calculation of PD).

The clinical relevance of the duration of stable disease varies in different studies and diseases. If the proportion of patients achieving stable disease for a minimum period of time is an endpoint of importance in a particular trial, the protocol should specify the minimal time interval required between two measurements for determination of stable disease.

## 10.4 NCI Common Terminology Criteria, Version 4.0

All relevant treatment centers should receive a copy of the NCI CTC AE version 4.0.

## 10.5 Effective treatment of EGFRTKI-related skin adverse events

In clinical practice, reasonable preventive measures and patient education are the most critical steps (see Section 6).

When skin damage occurs, the first step is to evaluate the extent of the lesion, and then processed step by step according to severity:

- - - **Mild:** patients may not need any form of intervention, can also use topical compound dexamethasone acetate, hydrocortisone (1% or 2.5% cream) or clindamycin (10% cream). Continue EGFR TKI at current dose, reassess rash after 2 weeks; if reactions worsen or do not improve, proceed to next step.
    - **Moderate:** topical 2.5% hydrocortisone ointment or erythromycin ointment, and oral ceramide, patients with symptoms should take oral minocycline as soon as possible (doxycycline); reassess rash after 2 weeks; if reactions worsen or do not improve, proceed to next step.
    - **Severe:** interventions are basically the same as moderate rashes, use strong dose of methylprednisolone and reduce EGFR TKI dose if necessary; if reactions do not improve after 2-4 weeks, dose interruption or discontinuation may be necessary.

## 10.6 Sample Analysis

The biopsy specimens and surgical pathology specimens for EGFR mutation detection during screening should submit in fresh tissue. The specimens should immediately ship to central laboratory for EGFR mutation detection during screening.

Blood sample collection time are shown as follows: 1 sample at pre- and post- neoadjuvant therapy, every 3 months in the first 2 years after surgery; if patients recurred 2 years after sugery, then taken blood sample once again at recurrence. Blood samples would be regularly sent to central laboratory for testing.

## 10.7 The New York Heart Association (NYHA) Functional Classification

|  | The New York Heart Association (NYHA) Functional Classification |
| --- | --- |
| **I** | No limitation of physical activity. Ordinary physical activity does not cause undue fatigue, palpitation, dyspnea (shortness of breath). |
| **II** | Slight limitation of physical activity. Comfortable at rest. Ordinary physical activity results in fatigue, palpitation, dyspnea (shortness of breath). |
| **III** | Marked limitation of physical activity. Comfortable at rest. Less than ordinary activity causes fatigue, palpitation, or dyspnea. |
| **IV** | Unable to carry on any physical activity without discomfort. Symptoms of heart failure at rest. If any physical activity is undertaken, discomfort increases. |

## 10.8 Calculation of creatinine clearance

**Female patients, Cockroft-Gault formula:**

Creatinine Clearance [ml/min]=

(140－age[yr]) × weight[kg]×0.85**)**/(72× serum Cr [mg/dl])

OR

(140－age[yr]) × weight[kg]×0.85**)**/(0.81×serum Cr [μmol/L])

**Male patients, Cockroft-Gault formula:**

Creatinine Clearance [ml/min]=

(140－age[yr]) × weight[kg]**)**/(72× serum Cr [mg/dl])

OR

(140－age[yr]) × weight[kg]**)**/(0.81×serum Cr [μmol/L])

**11. References**

[1] Ginsberg RJ, Goldberg M, Waters PE. Surgery in non-small lung cancer. In: Roth JA, Ruckdeschel JC, Weisenberger TH, editors. Thoracic Oncology. 2nd ed. Philadelphia: W.B. Saunders Company; 1995. p. 124-46.

[2] GLOBCAN 2002

[3] Rapp E, Pater JL, Willan A, Cormier Y, Murray N, Evans WK, et al. Chemotherapy can prolong survival in patients with advanced non-small-cell lung cancer--report of a Canadian multicenter randomized trial. J Clin Oncol 1988;6(4):633-41.

[4] van Meerbeeck JP, Surmont VF. Stage IIIA-N2 NSCLC: a review of its treatment approaches and future developments. Lung Cancer. 2009 Sep;65(3):257-67.

[5] Robinson LA, Ruckdeschel JC, Wagner H, Jr., Stevens CW. Treatment of non-small cell lung cancer-stage IIIA: ACCP evidence-based clinical practice guidelines (2nd edition). Chest. 2007 Sep;132(3 Suppl):243S-65S.

[6] van Meerbeeck JP, Kramer GW, Van Schil PE, Legrand C, Smit EF, Schramel F, et al. Randomized controlled trial of resection versus radiotherapy after induction chemotherapy in stage IIIA-N2 non-small-cell lung cancer. J Natl Cancer Inst. 2007 Mar 21;99(6):442-50.

[7] Albain KS, Swann RS, Rusch VW, Turrisi AT, 3rd, Shepherd FA, Smith C, et al. Radiotherapy plus chemotherapy with or without surgical resection for stage III non-small-cell lung cancer: a phase III randomised controlled trial. Lancet. 2009 Aug 1;374(9687):379-86.

[8] Sandler A. Clinical experience with the HER1/EGFR tyrosine kinase inhibitor celotinib. Oncology (Huntingt) 2003; 17:17-32.

[9]Perez-Soler R, Chachoua A, Hammond LA, et al. Determinants o f tumor response and survival with erlotinib in patients with non-small-cell lung cancer. J Clin Oncol 2004; 22:3238-3247.

[10] Miller VA, Patel J, Shah N, et al. The epidermal growth factor receptor tyrosine kinase inhibitor erlotinib (OSI-774) shows promising activity in patients with bronchioalveolar cell carcinoma (BAC): preliminary results of a phase II trial. Proc Am Soc Clin Oncol 2003; 22:619(abstract 2491).

[11] Shepherd FA, Rodrigues Pereira J, Ciuleanu T, et al. Erlotinib in previously treated non-small-cell lung cancer. N Engl J Med 2005; 353: 123-132.

[12] Reiter J L，Threadgill D W，Eley GD，et a1．Comparative genomic sequence analysis and isolation of human and mouse alternative EGFR transcripts encoding truncated receptor iso-fomls. [J]Genomics，2001，71(1)：1．

[13] Urich A，Coussens L，Hayrick J S，et a1．Human epidermal growth factor receptor eDNA sequence and aberrant expression of the amplified gene in A341 epidermoid carcinoma cells. [J] Nature，1984，309(5967)：418．

[14] Mayumi Ono，Michihiko Kuwano．Molecular Mechanisms of Epidermal Growth Factor Receptor (EGFR)Activation and Response to Gefitinib and Other EGFR—Targeting Drugs. [J] Clin Cancer Res，2006：24．

[15] Jiahong Wu，Qiuling Xie, Xiaojia Chen．Epidermal growth factor receptor and signal pathway. [J] Chinese Bulletin of Life Sciences，2006, 18(2)：116．

[16] Andriani Charpiani Chalpidou，Despoina Blarza，et a1．EGFR Mutations in Non-small Cell Lung Cancer．[J]Clinical Implications in vivo，2008，22：529．

[17] Rosell R, Moran T, Queralt C, et al. Screening for Epidermal Growth Factor receptor mutations in lung cancer. [J] N Engl J Med. 2009 Sep 3;361(10):958-67. Epub 2009 Aug 19.

[18] Mok TS, Wu YL, Thongprasert S, Yang CH, Chu DT, Saijo N, et al. Gefitinib or carboplatin-paclitaxel in pulmonary adenocarcinoma. N Engl J Med. 2009 Sep 3;361(10):947-57.

[19] Takamochi K, Suzuki K, Sugimura H, Funai K, Mori H, Bashar AH, et al. Surgical resection after gefitinib treatment in patients with lung adenocarcinoma harboring epidermal growth factor receptor gene mutation. Lung Cancer. 2007 Oct;58(1):149-55.

[20] Kappers I, Klomp HM, Burgers JA, Van Zandwijk N, Haas RL, van Pel R. Neoadjuvant (induction) erlotinib response in stage IIIA non-small-cell lung cancer. J Clin Oncol. 2008 Sep 1;26(25):4205-7.

[21] Wang Q, Wang H, Li P, Zhu H, He C, Wei B, et al. Erlotinib-Based Perioperative Adjuvant Therapy for a Case of Unresectable Stage IIIA (N2) Nonsmall Cell Lung Cancer. Am J Med Sci. 2010 Jul 1.

[22] Zhong W-Z, Yang X-N, Guo A-L, Chen H-J, Su J, Ri-Qiang L, et al. Induction erlotinib therapy in stage IIIA-N2 non-small cell lung cancer. Journal of Thoracic Oncology. 2009;4(9):S590-S1.

[23] Zhou C, Wu Y-l, Chen G, et al. Preliminary results of randomized phase Ⅲ study comparing

afficacy and safety of first -line erlotinib versus carboplatin (CBDCA) plus gemcitabine (GEM) in

Chinese advanced non-small cell lung cacer (NSCLC) patients (pts) with EGFR-activating

mutations (OPTIMAL) [abstract]. J Clin Oncol 2010;28 (Suppl 12 Pt I): 556s, 7575a.
